# Genetic Diversity and Identification of Vietnamese *Paphiopedilum* Species Using DNA Sequences

**DOI:** 10.3390/biology9010009

**Published:** 2019-12-31

**Authors:** Huyen-Trang Vu, Quoc-Luan Vu, Thanh-Diem Nguyen, Ngan Tran, Thanh-Cong Nguyen, Phuong-Nam Luu, Duy-Duong Tran, Truong-Khoa Nguyen, Ly Le

**Affiliations:** 1 Faculty of Biotechnology, Nguyen-Tat-Thanh University, 298A-300A Nguyen-Tat-Thanh Street, District 04, Hochiminh City 700000, Vietnam; vthtrang@ntt.edu.vn (H.-T.V.); 1511540870@ntt.edu.vn (T.-D.N.); Nguyencong0404@gmail.com (T.-C.N.);; 2 Faculty of Biotechnology, International University—Vietnam National University, Linh Trung Ward, Thu Duc District, Hochiminh City 700000, Vietnam; kimngan.tran@hdr.qut.edu.au; 3 Tay Nguyen Institute for Scientific Research, Vietnam Academy of Science and Technology, 116 Xo Viet Nghe Tinh, Ward 7, Da Lat City, Lam Dong province 66000, Vietnam; vuquocluan07@yahoo.com.vn; 4 Agricultural Genetics Institute, Pham Van Dong Street, Hanoi 100000, Vietnam; tdduong01@yahoo.com (D.-D.T.); ntkhoa128@yahoo.com (T.-K.N.)

**Keywords:** *Paphiopedilum*, molecular identification, species resolution, ITS, *mat*K, tree-based method

## Abstract

*Paphiopedilum* is among the most popular ornamental orchid genera due to its unique slipper flowers and attractive leaf coloration. Most of the *Paphiopedilum* species are in critical danger due to over-exploitation. They were listed in Appendix I of the Convention on International Trade in Endangered Species of Wild Fauna and Flora, which prevents their being traded across borders. While most *Paphiopedilum* species are distinctive, owing to their respective flowers, their vegetative features are more similar and undistinguished. Hence, the conservation of these species is challenging, as most traded specimins are immature and non-flowered. An urgent need exists for effective identification methods to prevent further illegal trading of *Paphiopedilum* species. DNA barcoding is a rapid and sensitive method for species identification, at any developmental stage, using short DNA sequences. In this study, eight loci, i.e., ITS, *LEAFY*, *ACO*, *mat*K, *trn*L, *rpo*B, *rpoC1,* and *trn*H-*psb*A, were screened for potential barcode sequences on the Vietnamese *Paphiopedilum* species. In total, 17 out of 22 *Paphiopedilum* species were well identified. The studied DNA sequences were deposited to GenBank, in which *Paphiopedilum dalatense* accessions were introduced for the first time. *ACO*, *LEAFY,* and *trn*H-*psb*A were limited in amplification rate for *Paphiopedilum*. ITS was the best single barcode. Single ITS could be used along with nucleotide polymorphism characteristics for species discrimination. The combination of ITS + *mat*K was the most efficient identification barcode for Vietnamese *Paphiopedilum* species. This barcode also succeeded in recognizing misidentified or wrongly-named traded samples. Different bioinformatics programs and algorithms for establishing phylogenetic trees were also compared in the study to propose quick, simple, and effective tools for practical use. It was proved that both the Bayesian Inference method in the MRBAYES program and the neighbor-joining method in the MEGA software met the criteria. Our study provides a barcoding database of Vietnamese *Paphiopedilum* which may significantly contribute to the control and conservation of these valuable species.

## 1. Introduction

*Paphiopedilum* is a beautiful and rare Slipper orchid genus. According to Averyanov et al. (2004) [1], there are 22 *Paphiopedilum* species, including four natural hybrids found in Vietnam (Table S1). In 2010, *Paphiopedilum canhii* was reported as a new species from Northern Vietnam. Then, the new subgenus *Megastaminodium* Braem & Gruss 2012 was published to accommodate *Paphiopedilum canhii* [2]. However, this species was immediately classified as being in danger, and has little chance of surviving in nature [3]. The species *Paphiopedilum armeniacum* is known to grow in Yunnan province, China. Although the presence of this pretty species in Vietnam is not recognized by official scientific reports, many collectors have detected their presence and distribution in limestone mountains in Vietnam at the northern border with China and Myanmar [4]. Although these 24 species are all accepted by the World Checklist of Selected Plant Families (https://wcsp.science.kew.org/qsearch.do), two hybrid species, *P. x affine* and *P. x aspesrum*, are data deficient and have seldom been found in Vietnam for years [1]. Therefore, in fact, the Vietnamese *Paphiopedilum* population consists of 22 species (Table S2).

Unfortunately, most Vietnamese *Paphiopedilum* are in critical of extinction [5], and some no longer exist in nature [1]. Uncontrolled sampling increases rapidly as a result of the coordination of local people and orchid traders in the collection of plants just days after the species were discovered. The conservation of populations in nature is very complicated. In addition to establishing protected areas and setting out regulations to prohibit illegal trade, customs and quarantine inspectors should have a basic understanding of plants, in order to be able to differentiate between rare and common species. However, most of the traded species are at the vegetative developmental stage, where there are high morphological similarities among species, leading to misidentification. Therefore, besides preliminary morphological determination [6], it is necessary to develop more effective identification methods for *Paphiopedilum* species.

DNA barcoding is a molecular identification method based on a short DNA sequence which is unique and representative of a species or a taxonomic level [7]. This quick and sensitive method allows researchers to successfully identify species with just a small amount of sample, making it efficient for plant conservation [8,9], especially at biodiversity hotspots [10,11,12] and for the discrimination of medicinal herbals from adulterants [13,14,15,16]. This approach has been widely applied using various sequence regions, of which ITS is the most used [17,18,19,20,21,22,23,24]. ITS2 was reported to have higher variability than full-length ITS, and can be an effective alternative [15,25]. The single *trn*H-*psb*A locus has also been widely evaluated in early studies, with a species resolution of up to 100% on a wide range of 72 angiosperm genera [26], or on a small range of species in one genus [27,28]. In addition, the combination of multiple loci of *trn*H-*psb*A, *rpo*B, *rpo*C1, and *mat*K has been recommended by many studies [19,29,30,31,32].

DNA barcoding was also applied to many taxa of Orchidaceae, such as *Dendrobium* [16,17,19,20,27,33,34,35], *Phalaenopsis* [36], *Cypripedium* [37], *Grammatophyllum* [18], *Cymbidium* [31], *Vanda* [38,39], and *Spathoglottis* [40]. For *Paphiopedilum*, the first barcoding research was of Parveen et al. (2012), on eight Indian species. Among five potential barcodes, i.e., *rpo*B, *rpo*C1, *rbc*L, *mat*K, and ITS, *mat*K was the most highly evaluated, with 100% species resolution, while ITS achieved only 50% [41]. However, in 2017, when these five regions were again applied on a large scale, i.e., 393 accessions of 94 Indian orchid species belonging to 47 genera, the authors found that the species resolution of *mat*K decreased (85.7%) due to denser sampling, while ITS was the best (94.9%) [42]. The two low-copy nuclear genes, *LEAFY* and *ACO*, were first used for Crypripedioideae, including *Paphiopedilum* species, by Guo et al. (2012) [43]. Although the sequences were not analyzed for barcoding but in order to study the evolutionary relationships, the reconstructed phylogenetic trees were reported to be relatively well-resolved and highly supported, showing potential for use as barcoding markers in *Paphiopedilum*. Guo et al. (2016) screened eight chloroplast loci, i.e., *rpo*C2, *atp*F-*atp*H, *ycf*1, *atp*I-*atp*H, *mat*K, *acc*D, *trn*S-*trn*fM, and *rbc*L, on 77 *Paphiopedilum* species in south-east Asia [44,45]. The species resolution of single- and multilocus of chloroplast barcodes was compared with ITS sequences from GenBank (https://www.ncbi.nlm.nih.gov/genbank/). The combination of *mat*K + *atp*F-*atp*H was the most efficient multilocus barcode, and was recommended for use with ITS. Although low-copy nuclear genes were not included in this study, the authors recommended the use of these regions in the DNA barcoding of this genus for more precise identification [45]. Recently, Rajaram et al. (2019) worked on four *Paphiopedilum* species of Peninsular Malaysia for discriminatory power using *rbc*L, *mat*K, ITS, and *trn*H-*psb*A loci. In that study, *mat*K was also shown to have the highest capability of resolution (100%) [28], as described by Parveen et al. (2012) [41].

In Vietnam, a phylogenetic study of Trung et al. (2013) was conducted on *Paphiopedilum* genus using a single ITS region [46]. Despite the fact that the tree showed 100% resolution, the study had a small sample size in which only 16 of the total 22 Vietnamese *Paphiopedilum* species, each with one single representative specimen, were included. As the sample size could significantly affect the resolution results [45,47,48,49], a larger number of samples, as well as additional discriminatory sequences, was recommended to develop a comprehensive identification method. The study of Trung et al. (2013) was the first in the construction of a molecular database of *Paphiopedilum* in Vietnam. So far, no other research has been carried out on barcoding the Vietnamese *Paphiopedilum* population.

In 2017, our group made a review of molecular markers for the identification of orchids from previous studies [49], and found that different resolution effects were obtained from different sequences used on different taxa. To examine the markers for the *Paphiopedilum* genus, we conducted an in silico study on 28 loci of *Paphiopedilum* species from GenBank sequences. *ACO*, *LEAFY*, ITS, *mat*K, and *trn*L were proposed as potential markers. Short ITS2 and full ITS were also compared, and ITS was again shown to be better [47]. In this study, we aimed to examine the species resolution of eight regions, i.e., ITS, *ACO*, *LEAFY*, *mat*K, *trn*L, *rpo*B, and *rpo*C1, for potential barcodes that are sufficient to identify Vietnamese *Paphiopedilum* species. We also suggested convenient bioinformatics tools for the practical use of barcoding technique in controling the trade of this genus.

## 2. Materials and Methods

### 2.1. Plant Materials

In total, 95 samples of 21 Vietnamese *Paphiopedilum* species (including 2 hybrid species) were obtained (Table S2). Two variants, *P. malipoense* var. *malipoense* and *P. malipoense* var. *jackii*, were treated as the same species. Attempts to collect samples of *Paphiopedilum canhii* species failed due to its critical endangered state. Except for two species, *P. vietnamense* and *P. herrmannii*, which had only one sample, at least three samples for each species were collected. Fresh leaves of specimens were obtained from the *Paphiopedilum* collections of the Tay Nguyen Institute for Scientific Research and the Agricultural Genetics Institute, Vietnam. Plants from this source had been collected from different areas of Vietnam and correctly identified by experts based on flower morphological description [1,3,50]. In addition, nine samples, i.e., ARM-41, CAL-166, CON-115, COC-150, COC-151, DEL-158T, TRA-177, TRA-178, and VIE-129, which came from trading sources were also included to confirm their scientific name based on this identification technique. Full scientific names, vernacular names, distribution areas, International Union for Conservation of Nature (IUCN) levels [5], and Vietnamese endemic list [1] are shown in Table S1.

### 2.2. DNA Extraction, Amplification, and Sequencing

Total DNA from fresh leaves was extracted using Isolate II Plant DNA kit BIO-52069 (TBR company, Ho Chi Minh City, Vietnam). The DNA was then stored at −20 °C and used as the template (100 ng per 50 uL reaction volume) for the amplification process. The thermal cycle was as follows: one cycle of DNA denaturation at 94 °C for 10 min, followed by 30 cycles of 30 s at 94 °C, 30 s at annealing temperature (Ta °C), and 40 s at 72 °C, with a final extension of 5 min at 72 °C, using SimpliAmp™ Thermal Cycler A24811 (Thermo Fisher Scientific Company, Waltham, MA USA). The Ta °C is different depending on the corresponding primer pairs. Details of Ta °C and primers used for the amplification of the ITS, *mat*K, *trn*L, *rpo*B, *rpo*C1, and *trn*H-*psb*A regions are shown in Table 1. Other primers for nuclear genes *ACO* and *LEAFY* are shown in Table S3, since neither the available nor newly-designed primers were effective in the study. The quality of all PCR products was checked using electrophoresis technique for the present of clear, unique band in agarose gel 1%. First, 40 uL volume of unpurified PCR product were sent directly to 1st BASE company (Singapore) for Sanger sequencing on both forward and reverse directions. The primers used for sequencing were the same as those in the PCR reactions (Table 1).

### 2.3. Collecting of Sequence Data

Dataset I: 23 samples of eight endemic *Paphiopedilum* species of Vietnam: *P. delenatii*, *P. x dalatense*, *P. gratrixianum*, *P. hangianum*, *P. helenae*, *P. x herrmannii*, *P. tranlienianum* and *P. vietnamense* (Table S2). Pilot testing was conducted on eight regions: ITS, *LEAFY*, and *ACO* from the nuclear genome, and *mat*K, *trn*L, *rpo*B, *rpoC1*, and *trn*H-*psb*A from the chloroplast genome.

Dataset II: Three regions, ITS, *mat*K and *trn*L, were amplified and sequenced on all 95 samples of 21 species from our sampling. For species *Paphiopedilum canhii*, available ITS and *mat*K accessions from the GenBank were added. In total, 22 species of the Vietnamese *Paphiopedilum* population were analyzed for species resolution. For each analysis, the closely-related Slipper species, *Phragmipedium longifolium*, was included as an outgroup. Details of the studied accession number are shown in Table S2.

### 2.4. Data Analysis

Raw sequences were trimmed off ambiguous ends before the consensus DNA sequence was created from forward and reverse sequences using FinchTV [52]. All the consensus sequences were submitted to the GenBank; their accession numbers are shown in Table S2. Alignments and nucleotide polymorphism measurements were managed automatically and manually using the Seaview software by Gouy et al. (2009) [53]. The genetic characteristics were calculated using MEGA7 [54]. The species resolution was estimated mostly based on the tree-based method, in combination with the polymorphism character-based method. For the tree-based discrimination technique, the monophyletic species were considered to have been successfully distinguished. For the species-specific SNP (Single Nulceotide Polymorphism) approach, unique variable-site characters can help to distinguish one species from the others.

Tree-based construction was carried out using different phylogenetic methods. The neighbor-joining (NJ) method was conducted using MEGA7 [54], and the Maximum Likelihood (ML) and Maximum Parsimony (MP) methods in the PAUP* 4.0 tool [55], as well as the Bayesian Inference (BA) method in the MRBAYES program (developed by John Huelsenbeck and Fredrik Ronquist in 2001) [56], Tree rooting was performed using the outgroup method. The nucleotide substitution models set up in each phylogeny running were inferred from the jModeltest program (developed by David Posadain 2008) [57]. The optimal models for ITS, *mat*K, *trn*L, and *mat*K + ITS were K80 + G, TIM1 + I, TPM1uf + I, and TPM1uf + G respectively. These proposed models for each DNA locus were applied to the PAUP* and MRBAYES programs. The model Kimura-2-parameters was used for MEGA analysis. Bootstrap 1000 was applied for reliability estimations. The tree-topology obtained from all phylogenetic running was visualized using the Figtree v1.4.3 program (developed by Andrew Rambaut in 2009) [58].

## 3. Results and Discussion

### 3.1. Identification Loci in Vietnamese Endemic Paphiopedilum Species

Firstly, a pilot screening of eight potential regions, i.e., ITS, *ACO*, *LEAFY*, *mat*K, *rpo*B, *rpoC1*, *trn*L, and *trn*H-*psb*A, was conducted, with priority being given to eight valuable endemic *Paphiopedilum* species of Vietnam (dataset I). Five regions ITS, i.e., *mat*K, *trn*L, *rpo*B, and *rpoC1*, were successfully amplified and sequenced on all studied samples, whereas *LEAFY* and *ACO* were hardly amplified, even with two different primer pairs; hence, there was no sequencing for those two regions. Therefore, ITS remained the best candidate as a nuclear locus for plant authentication. *trn*H-*psb*A could achieve 82.61% for amplification, but only 31.58% for sequencing (Table 2). *LEAFY*, *ACO,* and *trn*H-*psb*A were excluded from further sequence analyses.

**Figure 1:**
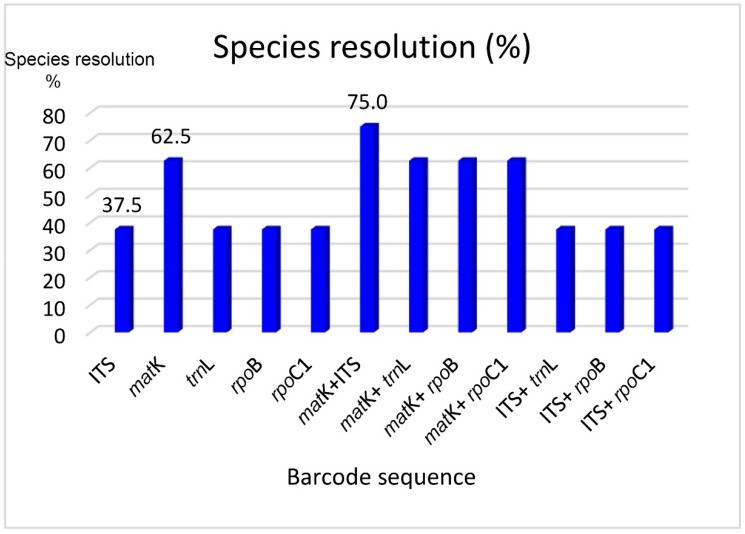
Species resolution results on eight *Paphiopedilum* endemic species (dataset I).

ITS, *mat*K, *trn*L, *rpo*B, and *rpoC1* all produced correct sequencing signals, and were therefore used for resolution analyses. The analysis mainly employed tree-based and genetic distance methods in the MEGA7 software with Kimura-2-Parameter (K2P) model and 1000 bootstrap replicates. The full trees are shown in Figure S1. Conspecific samples were identical and grouped on the same branch in all of the examined trees. Out of these loci, *mat*K gave the best resolution effect, with 5 out of 8 species being separated. The locus *rpo*B could just distinguish two species. ITS, *trn*L, and *rpoC1* showed the same rate, i.e., 3/8 (Table S4). The two-locus barcode combinations showed increasing capabilities. *mat*K + ITS had the best resolution, i.e., 75% (6/8 species) (Figure 1). The two nonseparated species were *Paphiopedilum gratrixianum* and *P. herrmannii* (Figure S1). However, these pilot results may be changed when a larger sample size is applied.

In terms of genetic distance (Figure S2), ITS had the highest average value, i.e., 0.041, followed by *trn*L (0.016) and *mat*K (0.013). The mean distances of *rpo*B and *rpoC1* were quite low, i.e., 0.008 and 0.005, respectively. Furthermore, the number of variable sites and indel fragments was highest for ITS, followed by *mat*K, *trn*L, *rpo*B, and *rpoC1* (Table 3). ITS included a large and highly-variable indel located at 181 bp to 257 bp, which is species-specific. Therefore, ITS, *mat*K, and *trn*L were chosen for the large-scale identification of the Vietnamese *Paphiopedilum* population.

*LEAFY* and *ACO* genes were first introduced by Guo et al. (2012) [43] as markers for investigating the evolutionary and biogeographical history of Slipper orchids, and were then proposed for us as DNA barcodes for the *Paphiopedilum* genus [45]. These two regions were reported to be even better than ITS in species discrimination in our previous in silico study [47] due to their significant genetic divergence. However, primers for the amplification of *ACO* and *LEAFY* proposed by Guo et al. (2012) contained ambiguous nucleotides, and so could be applied for a large range of subfamily Cyprideoideae including five genera, i.e., *Paphiopedilum*, *Cypripedium*, *Phragmipedium*, *Selenipedium*, and *Mexipedium*. Furthermore, the PCR products generated by these primers were too long (1853–3717 bp for *LEAFY* and 909–2178 bp for *ACO*) and could not be directly sequenced using the Sanger method (300–1000 bp). Hence, we designed new primer pairs (Table S3) based on available sequence data of *Paphiopedilum* for the specific amplification of these two loci, with expected product lengths ranging from 700–1000 bp [51]. However, those primers were inefficient, showing a 31.25% amplification rate (under 50%) after multiple repetitions and different annealing temperature tests (Table 2). Few and short conserved regions inside their nucleotide sequences led to difficulties in designing primers, as expected.

For *trn*H-*psb*A, although the amplification rate was 82.61% (Table 2), none of products could be obtained in the first reactions using the universal primers proposed by CBOL (2009) [32]. These primers seemed to be unsuitable for sequencing, as well when this rate was much lower, i.e., at 31.58% (Table 2). *trn*H-*psb*A is an intergenic spacer which contains many repeats and pseudogenes, coupled with a high rate of DNA mutation. This matter has also been mentioned in some previous publications [30,59]. Designing new primers to amplify this locus might solve few and short conserved-region problems, e.g., for *LEAFY* and *ACO*.

We tried to find available primers for the amplification of the *trn*L region. However, all *trn*L sequences of *Paphiopedilum* from the GenBank were submitted without accompanying published papers. Hence, we designed new primers from submitted sequence data [51]. Since *trn*L is not a highly-variable region, our new primers were well applied.

The chloroplast maturase K gene (*mat*K) was evaluated as a high variable coding region, and has been put forward as a core barcode for land plants. However, its low amplification rates, due to low universality, have been mentioned in many previous studies [19,21,60,61]. In a study by Parveen et al. (2012) on *Paphiopedilum*, the primer pair matK1F/matK1R was also unable to succeed 100%. We modified the two primers 56F [62] and 1326R [63] to develop new forward and reverse primers for *Paphiopedilum*. The results were much better, with a 100% amplification rate and fewer repeated reactions.

A potential barcode should be balanced between high divergence for high species resolution and a sufficiently conserved level for the design of universal primers. However, none of the single or combined barcodes could resolve 100% of all plant species. Therefore, developing specific primers for barcoding particular groups of plants is a solution worth considering. This pilot step, which reduced the time and cost, helped us to preliminarily select potential barcodes for large scale application. In summary, the nuclear region ITS and the two chloroplast regions, *mat*K and *trn*L, were used for further analyses on the large scale identification of the Vietnamese *Paphiopedilum* population.

### 3.2. Effects of Different Bioinformatic Tools on the Identification of the Vietnamese Paphiopedilum Population

In this analysis, all 94 collected samples of 21 Vietnamese *Paphiopedilum* species (Table S1) were identified using the tree-based method based on the three chosen regions, i.e., ITS, *mat*K, and *trn*L. Phylogenetic trees were constructed separately using different methods, i.e., Maximum Likelihood (ML), Maximum Parsimony (MP), neighbor-joining (NJ), and Bayesian Inference (BA). Complete trees are shown in Figures S3 and S4, in which the colorful taxa represented successfully-identified species as they clustered into one monophyletic branch. The black taxa indicates unidentified species. The species resolution results are summarized in Table 4.

**Figure 2:**
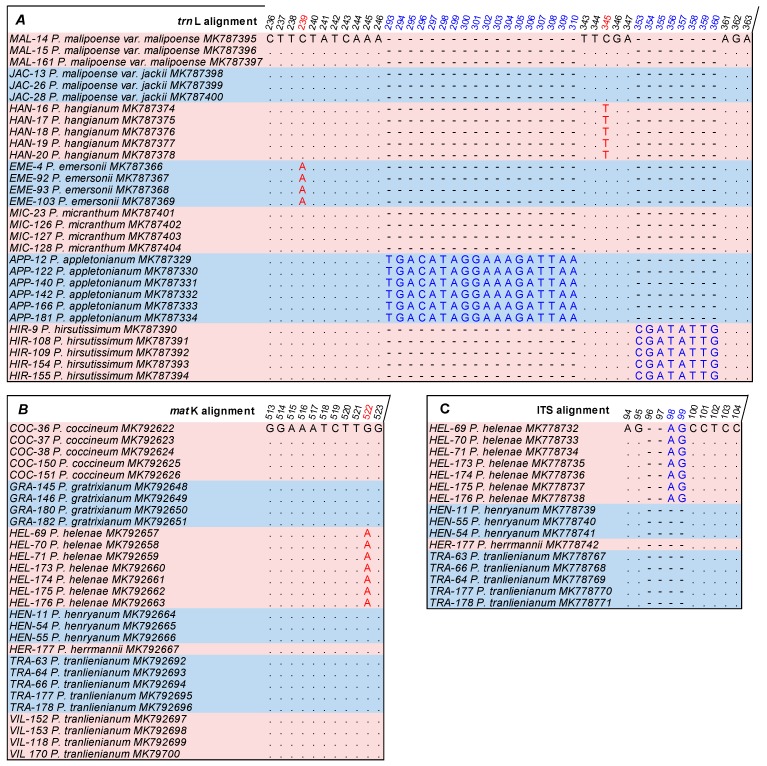
Informative variable sites from DNA alignment data of eight Vietnamese *Paphiopedilum* sequences on ITS, *mat*K, and *trn*L alignments using the MEGA software. (**A**): *trn*L alignment; (**B**): *mat*K alignment; (**C**): ITS alignment. (Number on top lines: alignment site; dot: nucleotide that is identical to one of the sequences at the first line; dash: gap; red color: site of nucleotide substitution; blue color: site of insertion fragment; fill color: was used to clarify the distinction between the sequences of the same species).

Single ITS gave the best resolution for 14 out of 21 species, followed by *mat*K (12 species) and *trn*L (6 species). In terms of species resolution, three methods, ML, NJ, and BA, gave the same identification results, while MP showed a slight difference on each of the three loci, ITS, *mat*K, and *trn*L (Table 4). To confirm the results, nucleotide polymorphism characteristics from alignment files were considered (Figure 2). For the MP tree, *P. appletonianum* accessions were not monophyletic by ITS (Figure S3D). However, based on ITS alignment, all conspecific sequences of *P. appletonianum* were 100% identical (data not shown). This result was totally in conformity with the ML, NJ, and BA trees, but not with the MP tree. By *mat*K, *P. helenae* accessions were also not monophyletic on the MP tree, but were in the group with six unidentified species, i.e., *P. coccineum*, *P. gratrixianum*, *P. henryanum*, *P. herrmannii*, *P. tranlienianum*, and *P. villosum* (Figure S4D). Nevertheless, the *mat*K data pointed out one substitution nucleotide A at site 359 in our alignment file (corresponding to nucleotide 522 of the complete *mat*K gene sequence from reference accession of *Paphiopedilum delenatii* MK463585.1) (Figure 2) that helps to distinguish this species from the others. Based on *trn*L, the two species, *P. emersonii* and *P. hangianum*, were set up in the same group with *P. malipoense* and *P. micranthum* in MP tree (Figure S5D). However, specific variable sites were found at nucleotides 152 and 258 in our alignment data (corresponding to the nucleotides 239 and 345 of the complete *trn*L gene sequence from the reference acession *Paphiopedilum delenatii* MK463585.1) (Figure 2). Hence, these two species should be separated from each others and from other species, *P. malipoense* and *P. micranthum*, as in ML, BA, and NJ trees (Figure S5). Hence, the difference in the results of Maximum Parsimony, which is based on the theory of the least character state changes [64], and three other methods in this study, was not supported. As a result, the use of the MP method for *Paphiopedilum* discrimination is not recommended.

From the alignment data, not only substitution variations but also indel information were useful. *P. helenae* was shown to contain an insertion of two nucleotides AG at site 93–94 in ITS alignment (corresponding to the nucleotides 98 and 99 of the complete ITS1 sequence from reference accession *Paphiopedilum delenatii* JQ660881.1) (Figure 2). This indel information showed that *P. helenae* sequences differ from the three species, i.e., *P. tranlienianum*, *P. herrmannii*, and *P. henryanum*, while the ITS trees (Figure S3) did not identify such a distinction. Therefore, a combination of phylogenetic tree and nucleotide polymorphism could help to identify more species. Moreover, we also detected two insertion fragments of *P. appletonianum* (nucleotides from 293 to 310) and *P. hirsutissimum* (nucleotides from 353 to 360) (Figure 2) which did not exist in other *Paphiopedilum* species sequences. Checking for the presence of these insertions in sequences might help to quickly recognize these species without other analyses. The use of indel information has been proposed [26,43,65] and efficiently applied [37,47] previously. Our study underlines the usefulness of this measurement in taxonomic research.

In terms of time efficiency, although we could not precisely present the duration of each tree-constructing process due to its dependence on the various computer configurations used, there were significant time differences among different programs. Here, we showed the relative period of tree construction with 1000 replications. The former number was based on the most straightforward dataset (*trn*L), and the latter on the most complex dataset (ITS) in our study. ML calculation using PAUP* took significant amounts of time (about 0.5–4 days), and hence, is not suitable for practical application at customs posts. We ran ML using the MEGA software and observed similar results to PAUP* in terms of resolution but at significantly higher speeds (about 10–30 min). Meanwhile, NJ run by MEGA took about 5–7 min, and MRBAYES took about 15–45 min. MRBAYES, which possesses more substitution models than MEGA and is faster than PAUP*, has also been noted as the best alternative to ML in phylogenetic tree building [66]. The examination of different bioinformatic approaches aimed to propose the most effective and convenient tools for the practical authentication of *Paphiopedilum*. The MEGA and MRBAYES programs seemed to meet the criteria.

### 3.3. Genetic Diversity and Identification of the Paphiopedilum Population of Vietnam

Six species, i.e., *P. dalatense*, *P. gratrixianum*, *P. henryanum*, *P. herrmannii*, *P. tranlienianum*, and *P. villosum,* were unidentified in all the examined trees. Despite not being separated, these six unknown species partitioned into two groups in the ITS tree, including the four-species group consisting of *P. tranlienianum*, *P. herrmannii*, *P. henryanum*, and *P. helenae*; and the three-species group consisting of *P. dalatense*, *P. gratrixianum,* and *P. villosum* (Figure S3). In contrast, in the *mat*K tree, *P. dalatense* was grouped with *P. appletonianum* and *P. callosum* but not *P. gratrixianum* and *P. villosum*, as in the ITS tree (Figure S4). Furthermore, *P. helenae,* which could not be distinguished by ITS data (Figure S3), was separated based on *mat*K (Figure S4). These results showed that a combination of ITS + *mat*K is promising in distinguishing among more species. Therefore, we combined ITS and *mat*K to evaluate the separation of Vietnamese *Paphiopedilum* species.

**Figure 3:**
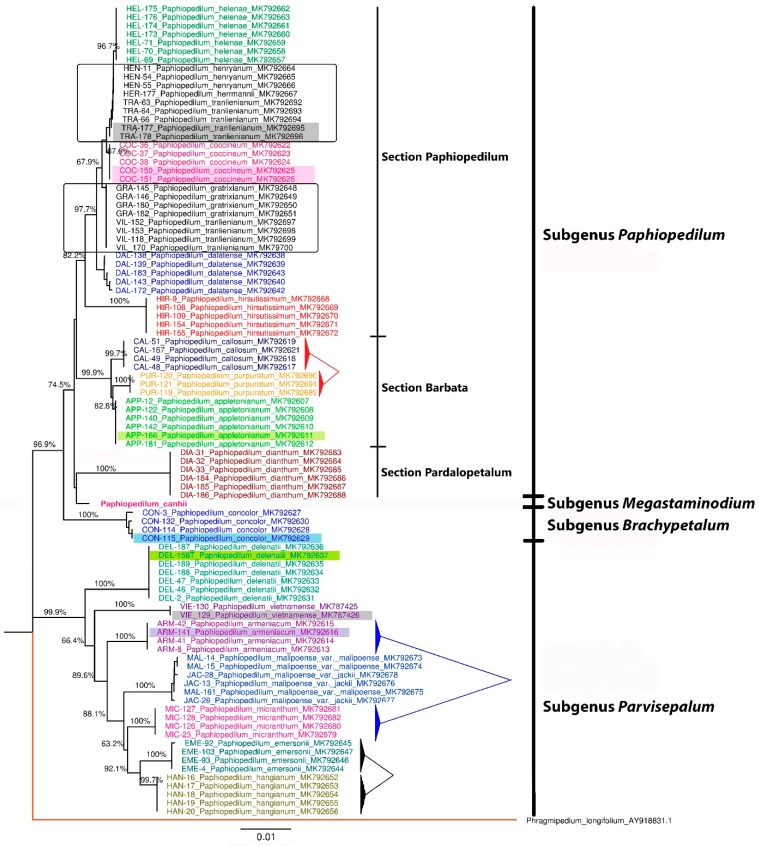
Phylogenetic tree of 22 Vietnamese *Paphiopedilum* species based on *mat*K + ITS barcode using the neighbor-joining method in the MEGA software. (Specimen voucher is before the scientific name, GenBank accession number is after the scientific name. *Phragmipedium longifolium* is the outgroup species. Five species in rectangular boxes and written in black are unidentified, the colored ones represent 17 identified species. Samples collected from trading markets were filled with highlighted colors. Three pairs of species, i.e., *P. emersonii* versus *P. hangianum*, *P. micranthum* versus *P. armeniacum*, *P. callosum* versus *P. purpuratum*, comprised leaf morphological similarity in pairs).

Phylogenetic trees based on ITS + *mat*K barcodes constructed in MEGA7 using the neighbor-joining method were representatively analyzed (Figure 3). A given species was considered to have been successfully identified when all of its accession grouped into a monophyletic branch which was not mixed with other species. Although there were still differences among conspecific accessions, as for *P. tranlienianum*, *P. dalatense*, *P. concolor,* and *P. malipoense*, no paraphyletic taxon was generated. *P. dalatense*, which was not idenfified by single ITS and *mat*K, could then be separated into an independent branch (Figure 3) on the ITS + *mat*K tree. This combination could indeed help to increase the number of identified species to 16 (Figure 3) instead of 14 in single ITS and 12 in single *mat*K (Table 4).

As no specimen of *Paphiopedilum canhii* species could be collected, we tried to collect its sequences from the reference GanBank for overall analysis. Two accessions of ITS and *mat*K were selected (shown in Table S2). No *trn*L accession was submitted. On the ITS + matK tree, *P. canhii* was located on a distinguished branch, and was quite far from its closest neighbor, *P. dianthum*. Due to this difference, *P. canhii* was classified in a new subgenus, *Megastaminodium* (Figure 3). In total, 17 out of the 22 species of the Vietnamese *Paphiopedilum* population, i.e., *P. helenae*, *P. coccineum*, *P. dalatense*, *P. hirsutissimum*, *P. callosum*, *P. purpuratum*, *P. appletonianum*, *P. dianthum*, *P. canhii*, *P. concolor*, P. *delenatii*, *P. vietnamense*, *P. armeniacum*, *P. malipoense*, *P. micranthum*, *P. emersonii*, and *P. hangianum*, were identified (Figure 3—colored taxa). All the monophyletic clusters were strongly supported with high bootstrap values.

The tree topology based on ITS + *mat*K data was divided into two main clusters. The first branch which included *P. delenatii*, *P. vietnamense*, *P. armeniacum*, *P. malipoense*, *P. micranthum*, *P. emersonii*, and *P. hangianum* corresponded with the subgenus *Parvisepalum* [1]. The second branch was divided into two main clusters which were also similar to the morphological system of Vietnamese *Paphiopedilum* species, as described by Averyanov et al. (2004) [1]. *P. concolor* corresponded with the subgenus *Brachypetalum*, and the other cluster was a member of the subgenus *Paphiopedilum*, which included three Sections: Paphiopedilum, Barbata, and Pardalopetalum (Figure 3).

The combination of two loci, ITS and *matK*, was proposed by Xu et al. (2015) on *Dendrobium* [17] and Xiang et al. (2011) on *Holcoglossum* orchids [21] as the best barcode after testing several combinations of two or three barcodes. Due to this multilocus combination, *P. dalatense* accessions which were not separated in both single ITS and *mat*K files were now grouped into a monophyletic branch. *P. dalatense* is a natural hybrid species of *P. callosum* and *P. villosum* [1,50]. This phenomenon explains why *P. dalatense* was grouped into the Paphiopedilum section, together with *P. villosum* in the ITS tree (Figure S3), and grouped into the Barbata section together with *P. callosum* in the *mat*K tree (Figure S4). The sequences of two varieties of *P. malipoense* were identical. This result was consistent with the observations of Trung et al. (2013) on some *Paphiopedilum* species of Vietnam [46]. Despite not being identified below the species level, these two variations, *P. malipoense* var. *malipoense* and *P. malipoense* var. *jackii*, could be recognized on the species level (Figure 3).

Although *mat*K was proposed as the best barcode, with 100% resolution in two previous *Paphiopedilum* studies [28,41], our study agreed with Parveen et al. (2017), who found that denser sampling decreased the resolution of *mat*K [42]. However, the combination of *mat*K with another locus was recommended as well. In an examination with other loci, i.e., *rpo*C2, *atp*F-*atp*H, ycf1, *atp*I-*atp*H, *acc*D, *trn*S-*trn*fM, and *rbc*L, it was suggested from a chloroplast sequence for *Paphiopedilum* by Guo et al. (2016) that *mat*K combined with *atp*F-*atp*H provided the best barcode (resolution 28.97%). Nevertheless, this combination was still shown to be lower than single ITS (52.27%). Hence, the authors proposed the three-locus combination of ITS, *mat*K, and *atp*F-*atp*H [45]. The more loci used, the more time and resources were required. In our study, it was recommended that *mat*K be directly combined with ITS, resulting in a resolution of 77.27%, higher than that reported by Guo et al. (2016). Furthermore, the two Vietnamese endemic species, *P. dalatense* and *P. herrmannii*, which were not mentioned in Guo et al. (2016) were first discussed here. There were some other different results between two studies. Two species, *P. henryanum* and *P. tranlienianum*, were identified in the study of Guo et al. study, but not in ours; the reason for this was the intraspecies diversity. Among the five specimens of *P. tranlienianum* in our sampling, two were different from *P. henryanum* at several nucleotides, while the other three were identical. Meanwhile, there was only one accession of *P. tranlienianum* in a study of Guo et al. (2017). We agreed that the higher resolution of our study might be because of the smaller sampling size, i.e., 22 species in comparison with 77 species in the study of Guo et al. (2017). However, since our study was applied in certain areas of Vietnam, 22 species in our collection could represent all the existing species in Vietnamese *Paphiopedilum* population. The presence of other *Paphiopedilum* species was limited. Hence, the application of the results from our study in the identification of Vietnam *Paphiopedilum* species was shown to be effective and practical.

### 3.4. Application in the Identification of Trading Paphiopedilum Samples

**Figure 4:**
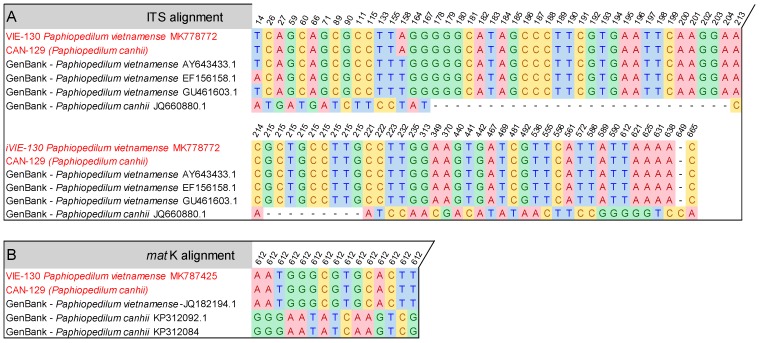
Nucleotide polymorphism of sample CAN-129 in comparison with referenced sequences from GenBank of *Paphiopedilum vietnamense* and *Paphiopedilum canhii* species. (**A**): alignment file using ITS sequences; (**B**): alignment file using *mat*K sequences. (Color accession: sample from our study. Black accession: reference accession from GenBank. Color of nucleotides represent four different nucleotides, i.e., A, T, G, C. Number above: site of variation on alignment data).

Besides the samples collected from scientific research institutes, nine samples were collected from trading markets, i.e., APP-166, ARM-41, CAN-129, CON-115, COC-150, COC-151, DEL-158T, TRA-177, and TRA-178 (Figure 3—Highlighted taxa). These samples were identified using the barcodes suggested in our study. Among them, a sequence of sample ARM-41 matched with accessions of *P. armeniacum*, CON-115 with *P. concolor*, COC-150 and COC-151 with *P. coccineum*, and TRA-177 and TRA-178 with *P. tralienianum*. Specimen *P. callosum* CAL-166, however, was not grouped with other *P. callosum* accessions, but with *P. appletonianum* sequences (Figure 4). Therefore, this sample was corrected for the scientific name *Paphiopedilum appletonianum* APP-166 when submitted to National Centre for Biotechnology Information (NCBI). DEL-158T is a white-flower variety of the *Paphiopedilum delenatii* species. The result showed that its sequences were identical to the original pink-flowered species (Figure 3). These results again confirmed the use of these loci for barcoding *Paphiopedilum* at the species level.

In our sampling, one commercial sample with the vernacular name Hai Xuan Canh was collected. We named it “voucher CAN-129”, with the expected scientific name *P. canhii*. However, this sample grouped with *P. vietnamense* VIE-130 in the phylogenetic tree (Figure 3). Its sequences were also 100% identical with *P. vietnamense* VIE-130 in all ITS, *mat*K, and *trn*L alignment data. To check its identity, we aligned its sequences with both *P. vietnamense* and *P. canhii* sequences from GenBank. No GenBank accession of *trn*L was found, and as such, we could not perform a comparison with this locus. There were 84 variable sites inside the ITS alignment and 15 substitution variations in the *mat*K alignment (Figure 4). Notably, sequences of CAN-129 were significantly different from GenBank *P. canhii*, but highly similar to those of GenBank *P. vietnamense* in both ITS and *mat*K data. The analysis confirmed the homology of this specimen with *P. vietnamense*. The name of the sample was corrected to *Paphiopedilum vietnamense* VIE-129 in the NCBI database. In practice, misidentification or confusion among species usually happens due morphologically similar leaves, or the young leaves of immature plants. The results again affirm the role of the sequence method on the accurate identification of species in nature.

### 3.5. The Support of Molecular Characters for Morphological Features

**Figure 5:**
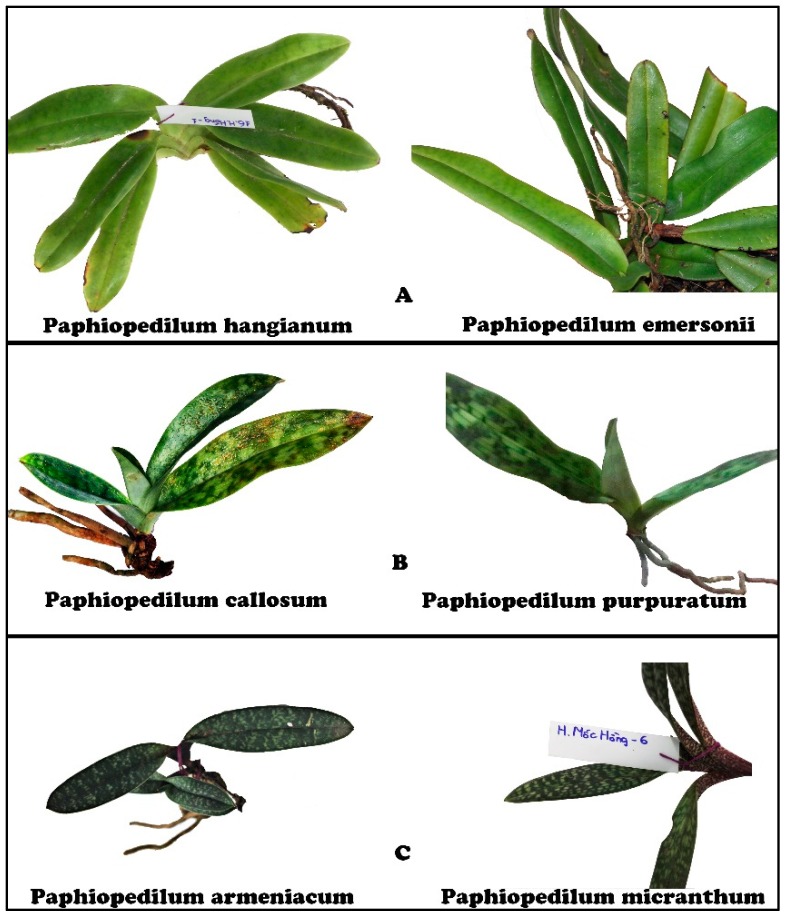
Similar leaf morphology in pairs of *Paphiopedilum* species. (**A**) *P. hangianum* versus *P. emersonii*, (**B**) *P. callosum* versus and *P. purpuratum*, (**C**) *P. armeniacum* versus *P. micranthum.*

Morphology-based methods are more time- and cost-effective than molecular identification techniques. However, to *Paphiopedilum* and some land plants, these methods are based mainly on indistinguishable reproductive parts, which reduces hinders their effectiveness. As for leaves and roots, there are few species-specific morphological characteristics, and hence, leaf- or root-based discrimination often leads to misidentification between similar entities. Examples of such objects in the *Paphiopedilum* genus are *P. hangianum* versus *P. emersonii*, *P. callosum* versus *P. purpuratum*, and *P. armeniacum* versus *P. micranthum* (Figure 5). Both *P. hangianum* and *P. emersonii* have large, hard, and thick leaves. Their leaves are uniformly green on both sides. whereas those of both *P. callosum* and *P. purpuratum* are the same in both size and shape. Both of their leaves are clearly mottled on the upperside and plain light green on the lowerside. For the pair, *P. armeniacum* and *P. micranthum*, dark green mottles on the upperside and dense purple dots on the lowerside are homologous features that make it difficult to distinguish between the two [6].

In this study, we successfully separated all of these pairs of species according to phylogenetic trees (Figure 3). Using molecular sequences, *P. emersonii* and *P. hangianum* were separated into two monophyletic clades with high support, i.e., 92.1%. *P. micranthum* had a closer relationship to the group of *P. emersonii* and *P. hangianum* than to *P. armeniacum*. *P. purpuratum* was similar to *P. appletoniaum* rather than to *P. callosum*, at 99.9% reliability. Therefore, molecular and morphological methods can be used in combination for significant increases in species resolution.

## 4. Conclusions

In practical conservation, the ability to quickly and accurately identify species is crucial. DNA sequencing can be effectively used for this purpose. From eight examined loci, we recommend the use of combined ITS + *mat*K as the most effective method for the molecular identification of Vietnamese *Paphiopedilum* species. Single ITS was also used in combination with nucleotide polymorphism to increase the species resolution. Using this approach, 17 out of 22 species of Vietnam *Paphiopedilum* populations were identified. Unidentified species were divided into two small groups: one comprised *P. gratrixianum* and *P. villsoum*, and other *P. henryanum*, *P. herrmannii,* and *P. tranlienianum*. A great deal of software and a number of tools have also been developed for different targets from laboratory research to practical applications. The neighbor-joining method using the MEGA software is both simple and effective for the barcoding of targets. Molecular techniques can be used alone or as a support for leaf-morphology in the classification of *Paphiopedilum* species.

Our research results contribute to the conservation and control of the illegal trade of *Paphiopedilum* species in Vietnam. As the species resolution was not able to achieve 100% accuracy, more work should be undertaken. Primer designing for other highly-variable regions is one avenue that needs to be examined.

## Figures and Tables

**Table 1:** Primers used for amplification reactions in the study.

Locus	Annealing Temperature (°C)	Primer Name	Primer Sequence	Expected Product Length (bp)	Reference
ITS	58	IT1–F	5′-AGTCGTAACAAGGTTTCC-3′	900	[24]
		IT2–R	5′-GTAAGTTTCTTCTCCTCC-3′		
*mat*K	55	F56–mo	5′-CCTATCCATCTGGAAATCTTAG-3′	1200	[51]
		R1326–mo	5′-GTTCTAGCACAAGAAAGTCG-3′		
*trn*L	62	*trn*L–F	5′-GGTAGAGCTACGACTTGATT-3′	600	
		*trn*L–R	5′-CGGTATTGACATGTAAAATGGGACT-3′		
*rpo*B	53	2F	5′-ATGCAACGTCAAGCAGTTCC-3′	600	[32]
		4R	5′-GATCCCAGCATCACAATTCC-3′		
*rpo* *C1*	53	1F	5′-GTGGATACACTTCTTGATAATGG-3′	600	
		3R	5′-TGAGAAAACATAAGTAAAGGGC-3′		
*trn*H-*psb*A	53	*psb*A3′f	5′-CGCGCATGGTGGTTCACAATCC-3′	900	
		*trn*Hf	5′-GTTATGCATGAACGTAATGCTC-3′		

**Table 2:** Amplification and sequencing results of eight loci on eight *Paphiopedilum* endemic species (dataset I).

No.	Species	Specimen Voucher	*ACO*		*LEAFY*		ITS	*mat*K	*trn*L	*rpo*B	*rpo*C1	*trn*H-*psb*A
			ACO F1/R1	ACO F2/R2	LFY F1/R1	LFY F2/R2						
1	*Paphiopedilum delenatii*	DEL-2	−/	−/	−/	−/	+/+	+/+	+/+	+/+	+/+	+/−
		DEL-46	−/	−/	−/	−/	+/+	+/+	+/+	+/+	+/+	+/−
		DEL-47					+/+	+/+	+/+	+/+	+/+	+/−
		DEL-187					+/+	+/+	+/+	+/+	+/+	+/−
		DEL-188					+/+	+/+	+/+	+/+	+/+	−/
2	*Paphiopedilum x dalatense*	DAL-138	−/	−/	−/	−/	+/+	+/+	+/+	+/+	+/+	+/+
		DAL-139	−/	−/	−/	−/	+/+	+/+	+/+	+/+	+/+	+/+
		DAL-143	−/	−/	−/	−/	+/+	+/+	+/+	+/+	+/+	+/−
3	*Paphiopedilum gratrixianum*	GRA-145	−/	−/	−/	−/	+/+	+/+	+/+	+/+	+/+	+/+
		GRA-146	−/	−/	−/	−/	+/+	+/+	+/+	+/+	+/+	+/−
		GRA-180					+/+	+/+	+/+	+/+	+/+	+/−
		GRA-182					+/+	+/+	+/+	+/+	+/+	+/−
4	*Paphiopedilum hangianum*	HAN-16	−/	−/	−/	−/	+/+	+/+	+/+	+/+	+/+	+/−
		HAN-17	+/	−/	−/	−/	+/+	+/+	+/+	+/+	+/+	+/−
		HAN-18	−/				+/+	+/+	+/+	+/+	+/+	+/−
5	*Paphiopedilum helenea*	HEL-69	−/	−/	−/	−/	+/+	+/+	+/+	+/+	+/+	−/
		HEL-70	−/	−/	−/	−/	+/+	+/+	+/+	+/+	+/+	+/−
		HEL-71					+/+	+/+	+/+	+/+	+/+	+/−
6	*Paphiopedilum x herrmannii*	HER-177	−/	−/	−/	−/	+/+	+/+	+/+	+/+	+/+	−/
7	*Paphiopedilum tranlienianum*	TRA-63	+/	−/	−/	−/	+/+	+/+	+/+	+/+	+/+	+/+
		TRA-64	+/	−/	−/	−/	+/+	+/+	+/+	+/+	+/+	+/+
		TRA-66	+/				+/+	+/+	+/+	+/+	+/+	+/+
8	*Paphiopedilum vietnamense*	VIE-130	+/	−/	−/	−/	+/+	+/+	+/+	+/+	+/+	−/
Number of successful amplification			5/16	0/14	0/14	0/14	23/23	23/23	23/23	23/23	23/23	19/23
**Rate of successful amplification**			**31.25%**	**0%**	**0%**	**0%**	**100%**	**100%**	**100%**	**100%**	**100%**	**82.61%**
Number of successful sequencing							23/23	23/23	23/23	23/23	23/23	6/19
**Rate of successful sequencing**							**100%**	**100%**	**100%**	**100%**	**100%**	**31.58%**

*ACO*, *LEAFY*, ITS, *mat*K, *trn*L, *rpo*B, *rpoC1*, and *trn*H-*psb*A were the studied regions. F1/R1, F2/R2 were different primer pairs for each region of *ACO* and *LEAFY*. Blank cell: no study. Signs +/−. The former sign is amplification result, the latter the sequencing result. Plus is a successful result, minus is a failed result.

**Table 3:** Genetic characteristics of ITS, *mat*K, *trn*L, *rpo*B, and *rpoC1* sequences of eight *Paphiopedilum* species.

Sequence Locus	Alignment Length (bp) (L)	Parsimony Site (P)	Singleton Site (S)	Variable Site (V = P + S)	Variable Rate (%) (V/L)	Indel Fragment
ITS	725	166	71	237	32.7	20
matK	1132	74	39	113	10.0	1
*trn*L	466	25	9	34	7.3	3
*rpo*B	483	7	5	12	2.5	0
*rpo*C1	460	5	3	8	1.7	0

**Table 4:** Species resolution of different barcodes on Vietnamese *Paphiopedilum* populations using different bioinformatic tools in different software.

		ITS					*mat*K					*trn*L				
No.	SPECIES	NJ	ML1	ML2	MP	BA	NJ	ML1	ML2	MP	BA	NJ	ML1	ML2	MP	BA
1	*Paphiopedilum appletonianum*	+	+	+	−	+	−	−	−	−	−	−	−	−	−	−
2	*Paphiopedilum armeniacum*	+	+	+	+	+	+	+	+	+	+	+	+	+	+	+
3	*Paphiopedilum callosum*	+	+	+	+	+	−	−	−	−	−	−	−	−	−	−
4	*Paphiopedilum coccineum*	+	+	+	+	+	−	−	−	−	−	−	−	−	−	−
5	*Paphiopedilum concolor*	+	+	+	+	+	+	+	+	+	+	−	−	−	−	−
6	*Paphiopedilum dalatense*	−	−	−	−	−	−	−	−	−	−	−	−	−	−	−
7	*Paphiopedilum delenatii*	+	+	+	+	+	+	+	+	+	+	+	+	+	+	+
8	*Paphiopedilum dianthum*	+	+	+	+	+	+	+	+	+	+	+	+	+	+	+
9	*Paphiopedilum emersonii*	+	+	+	+	+	+	+	+	+	+	+	+	+	−	+
10	*Paphiopedilum gratrixianum*	−	−	−	−	−	−	−	−	−	−	−	−	−	−	−
11	*Paphiopedilum hangianum*	+	+	+	+	+	+	+	+	+	+	+	+	+	−	+
12	*Paphiopedilum helenea*	−	−	−	−	−	+	+	+	−	+	−	−	−	−	−
13	*Paphiopedilum henryanum*	−	−	−	−	−	−	−	−	−	−	−	−	−	−	−
14	*Paphiopedilum herrmannii*	−	−	−	−	−	−	−	−	−	−	−	−	−	−	−
15	*Paphiopedilum hirsutissimum*	+	+	+	+	+	+	+	+	+	+	+	+	+	+	+
16	*Paphiopedilum malipoense*	+	+	+	+	+	+	+	+	+	+	−	−	−	−	−
17	*Paphiopedilum micranthum*	+	+	+	+	+	+	+	+	+	+	−	−	−	−	−
18	*Paphiopedilum purpuratum*	+	+	+	+	+	+	+	+	+	+	−	−	−	−	−
19	*Paphiopedilum tranlienianum*	−	−	−	−	−	−	−	−	−	−	−	−	−	−	−
20	*Paphiopedilum vietnamense*	+	+	+	+	+	+	+	+	+	+	−	−	−	−	−
21	*Paphiopedilum villosum*	−	−	−	−	−	−	−	−	−	−	−	−	−	−	−
**Number of monophyletic species**		**14**	**14**	**14**	**13**	**14**	**12**	**12**	**12**	**11**	**12**	**6**	**6**	**6**	**4**	**6**

(ITS, *mat*K, and *trn*L were single barcodes. NJ: neighbor-joining method in the MEGA software, ML1: Maximum Likelihood method in the PAUP* software, ML2: Maximum Likelihood in the MEGA software, MP: Maximum Parsimony in the PAUP* software, BA: Bayesian Inference in the MRBAYES software. Plus in blue cell: successfully-identified species. Minus in white cell: unidentified species).

## Supplementary Materials

The following are available online at https://www.mdpi.com/2079-7737/9/1/9/s1, Figure S1. Phylogenetic trees of 8 endemic *Paphiopedilum* species based on single and 2-locus combined sequences of ITS, *mat*K, *trn*L, *rpo*B, *rpo*C1. Figure S2. Estimates of evolutionary divergence between sequences of 8 endemic *Paphiopedilum* species based on 5 single sequences ITS, *mat*K, *trn*L, *rpo*B, *rpo*C1. Figure S3. Phylogenetic trees of 21 Vietnamese *Paphiopedilum* species based on ITS region using different methods. Figure S4. Phylogenetic trees of 21 Vietnamese *Paphiopedilum* species based on *mat*K region using different methods. Figure S5. Phylogenetic tree of 21 Vietnamese *Paphiopedilum* species based on *trn*L region using different methods. Table S1. Full scientific name, vernacular name, distribution areas, IUCN Red List state and endemic list of Vietnamse *Paphiopedilum* species.Table S2. Specimen voucher, collected source and accession numbers of studied *Paphiopedilum* samples.Table S3. Primers for screening of *ACO* and *LEAFY* in the study.Table S4. Species resolution results on 8 *Paphiopedilum* endemic species (dataset I).
