# Supplementary material for: Genetic Diversity and Identification of Vietnamese Paphiopedilum Species Using DNA Sequences
Source: Biology (Basel). 2019 Dec 31;9(1):9. doi: 10.3390/biology9010009 (PMC7168009; doi:10.3390/biology9010009)
Supplement: Supplementary file 1 [file biology-09-00009-s001.zip › Supplementary Figure S1.pdf]

Figure S1. Phylogenetic trees of 8 endemic *Paphiopedilum* species based on single and 2-locus combined sequences of ITS, *mat*K, *trn*L, *rpo*B, *rpo*C1. (**A**) ITS; (**B**)*mat*K; (**C**) *trn*L; (**D**) *rpo*B; (**E**) *rpo*C1; (**F**) *mat*K + ITS; (**G**) *mat*K + *trn*L; (**H**) *mat*K+ *rpo*B; (**I**) *mat*K + *rpo*C1, (**J**) ITS + *trn*L; (**K**) ITS + *rpo*B; (**L**) ITS + *rpo*C1

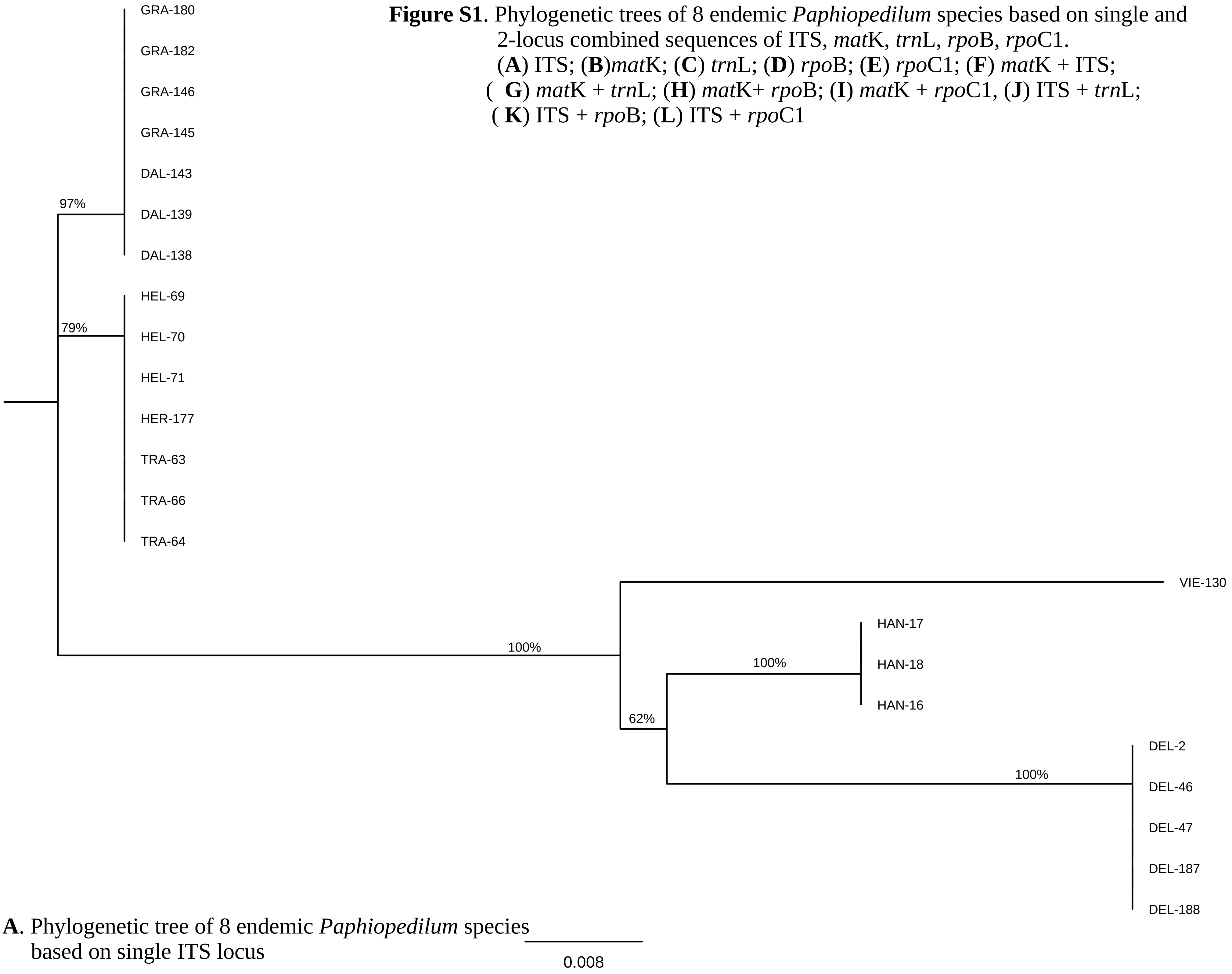

A. Phylogenetic tree of 8 endemic *Paphiopedilum* species based on single ITS locus

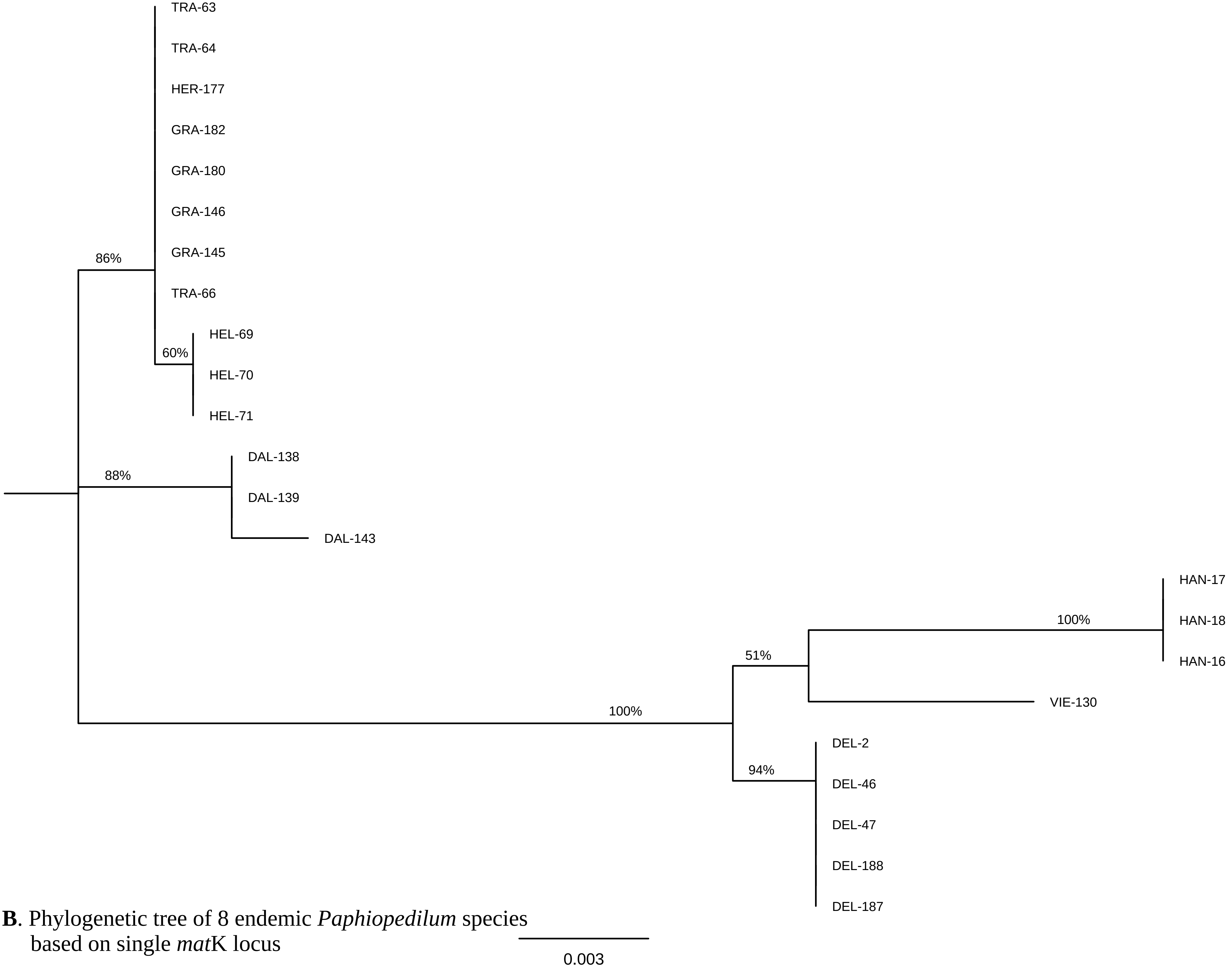

B. Phylogenetic tree of 8 endemic *Paphiopedilum* species based on single *mat*K locus

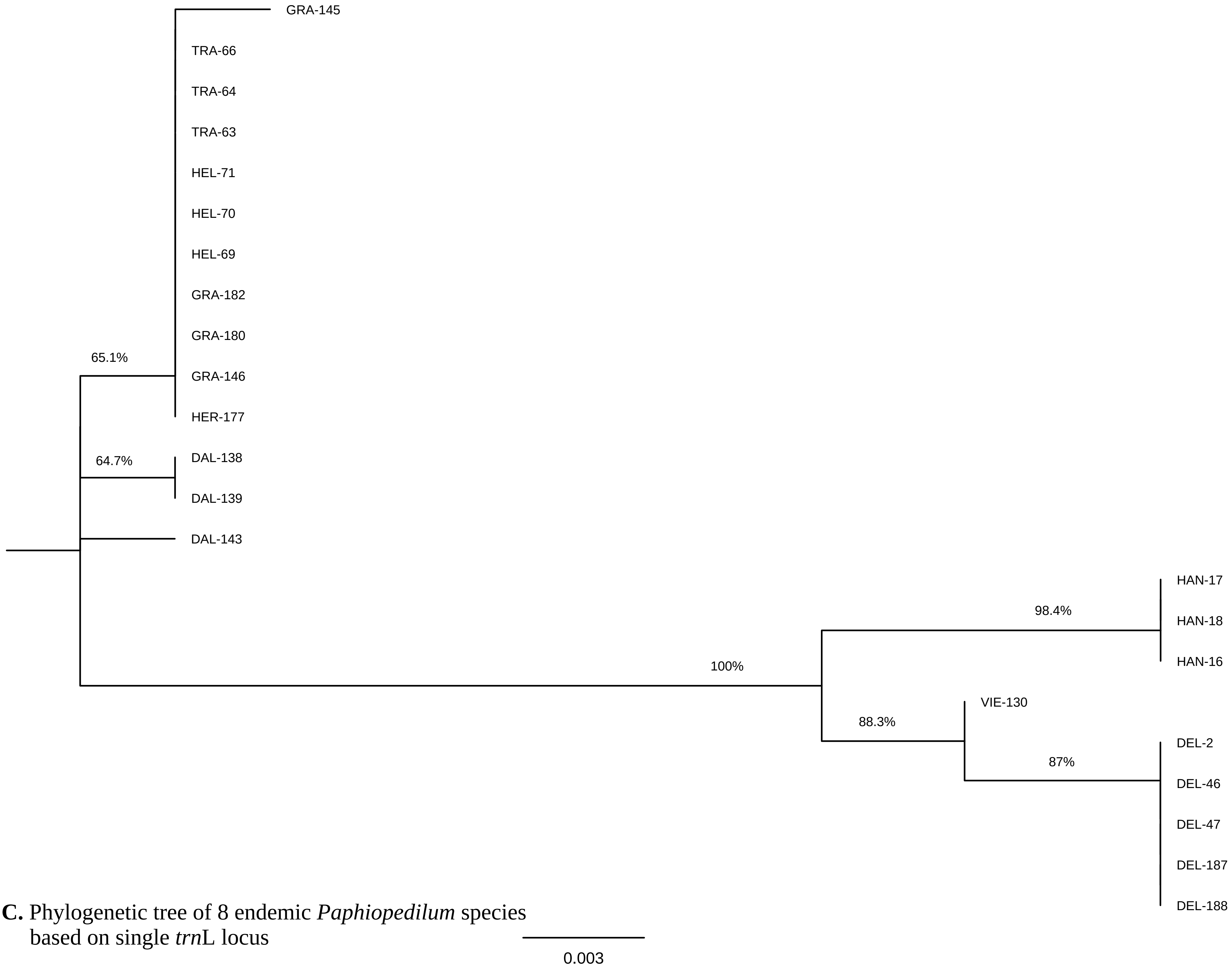

C. Phylogenetic tree of 8 endemic *Paphiopedilum* species based on single *trn*L locus

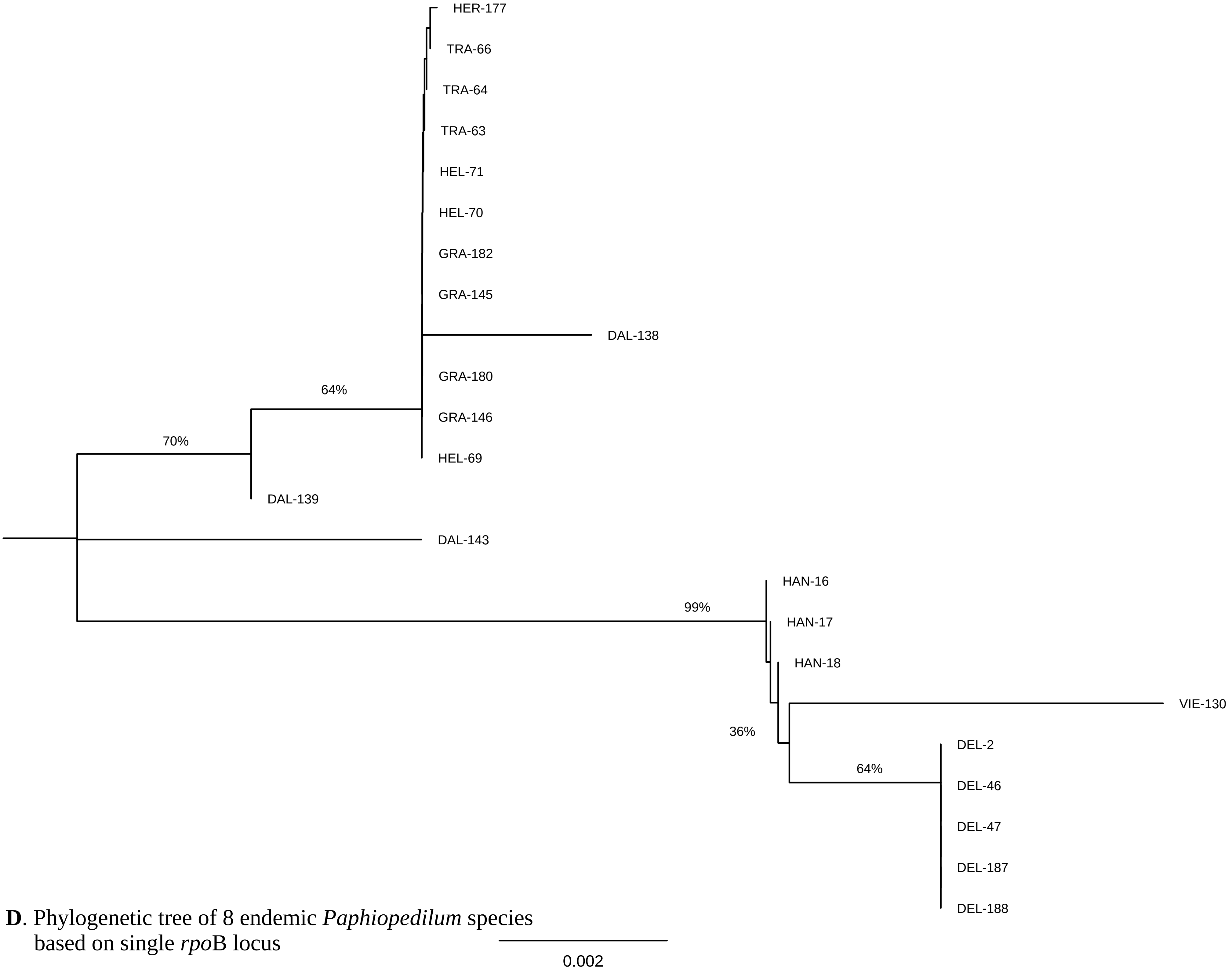

D. Phylogenetic tree of 8 endemic *Paphiopedilum* species based on single *rpo*B locus

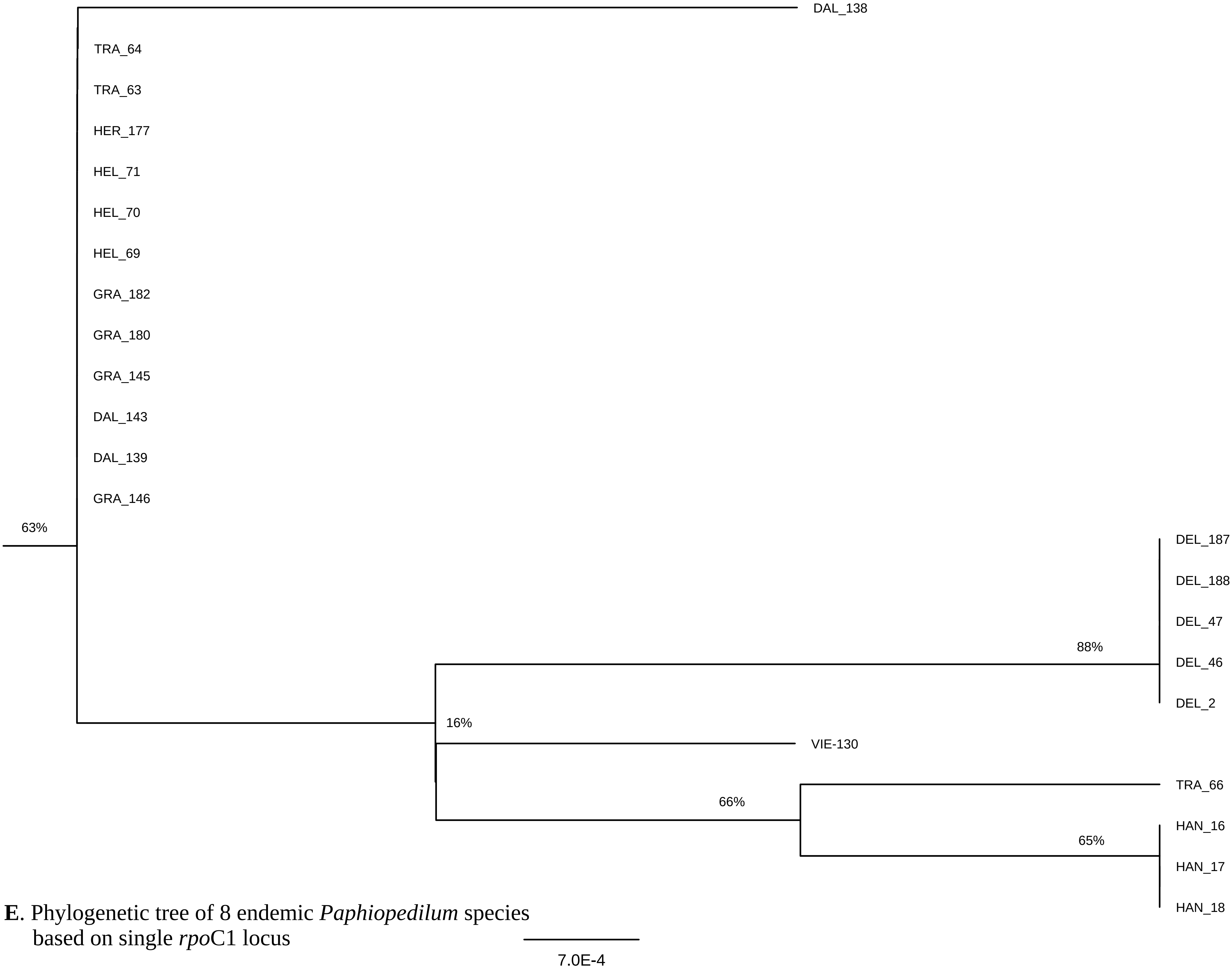

E. Phylogenetic tree of 8 endemic *Paphiopedilum* species based on single *rpo*C1 locus

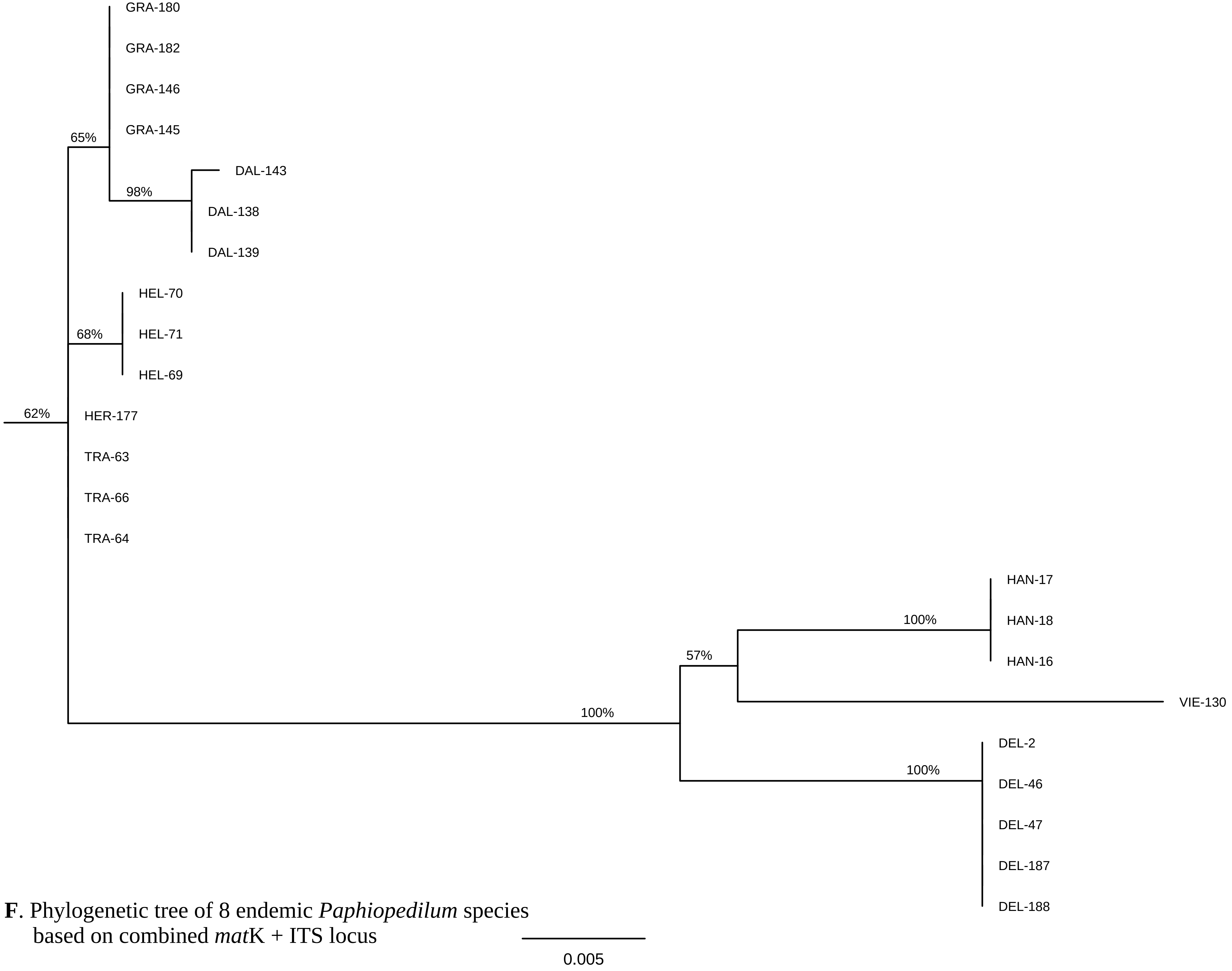

F. Phylogenetic tree of 8 endemic *Paphiopedilum* species based on combined *mat*K + ITS locus

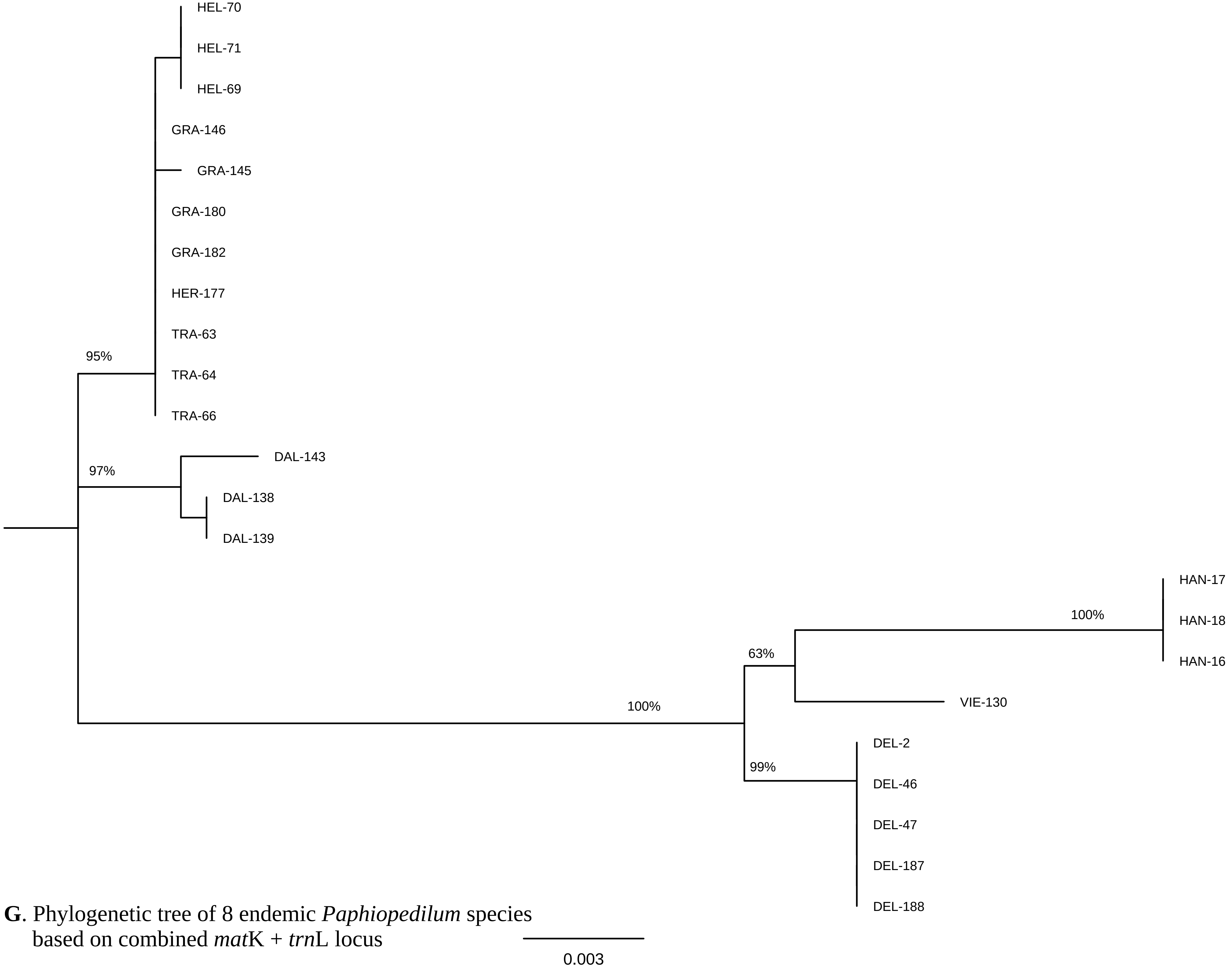

G. Phylogenetic tree of 8 endemic *Paphiopedilum* species based on combined *mat*K + *trn*L locus

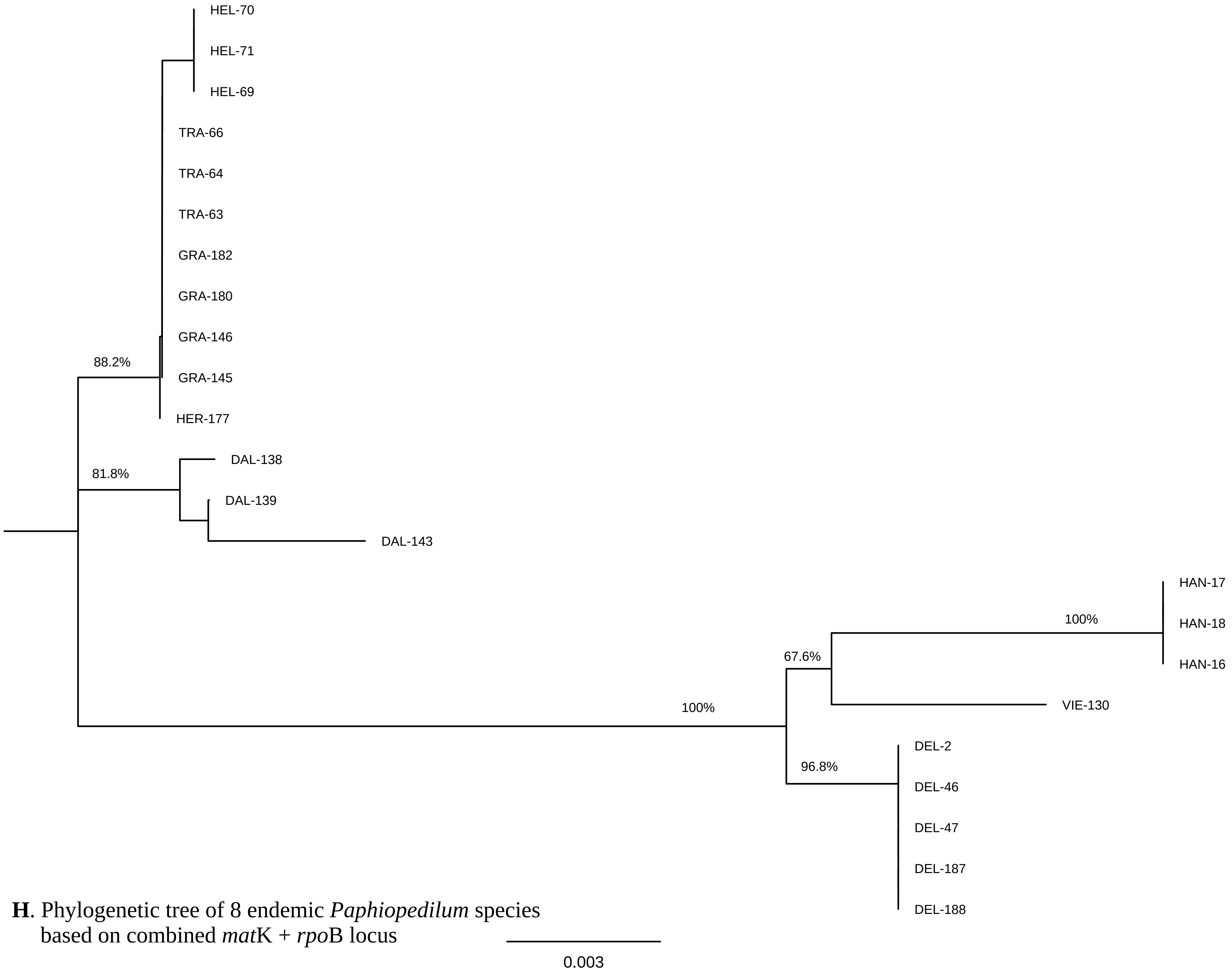

H. Phylogenetic tree of 8 endemic *Paphiopedilum* species based on combined *mat*K + *rpo*B locus

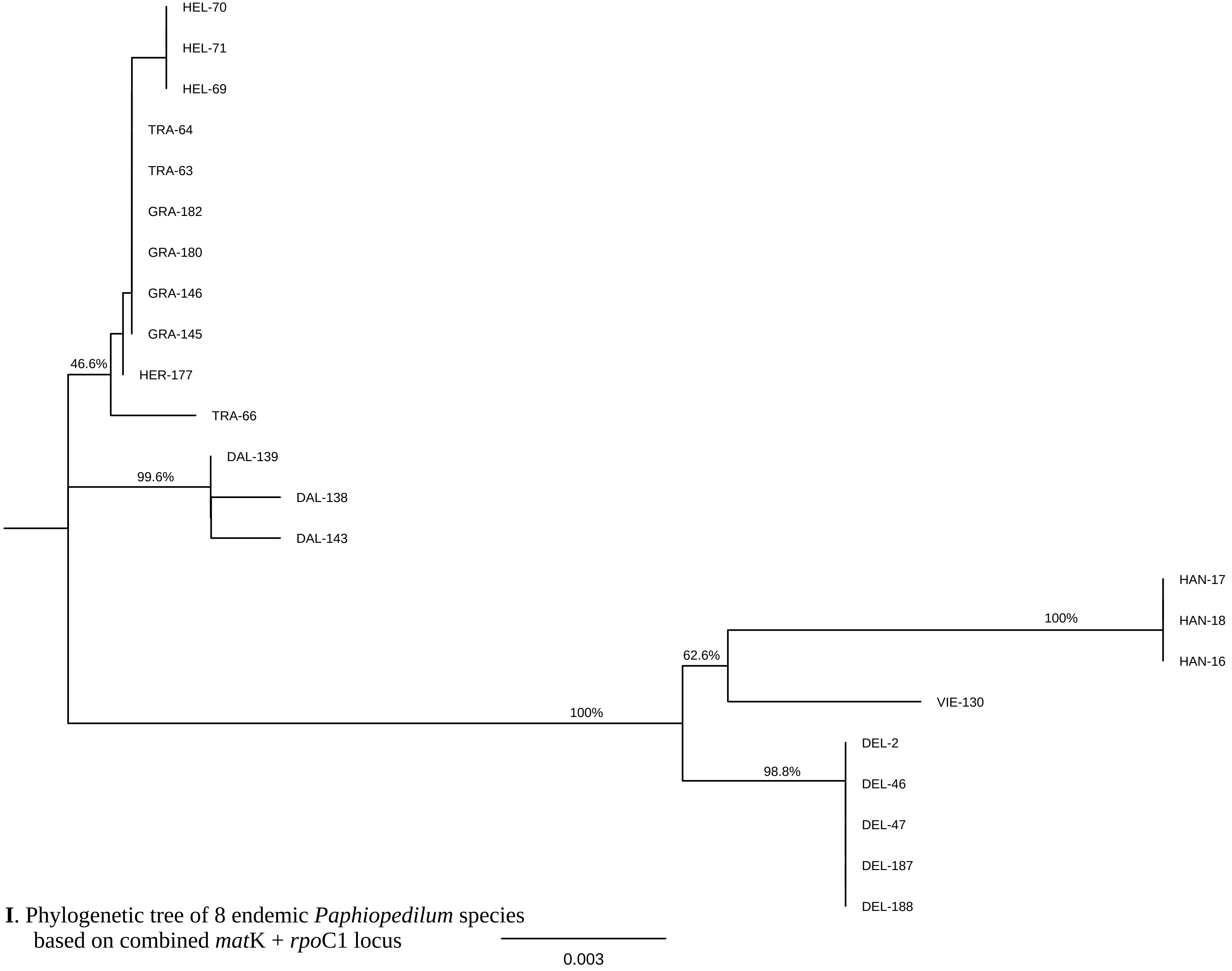

I. Phylogenetic tree of 8 endemic *Paphiopedilum* species based on combined *mat*K + *rpo*C1 locus

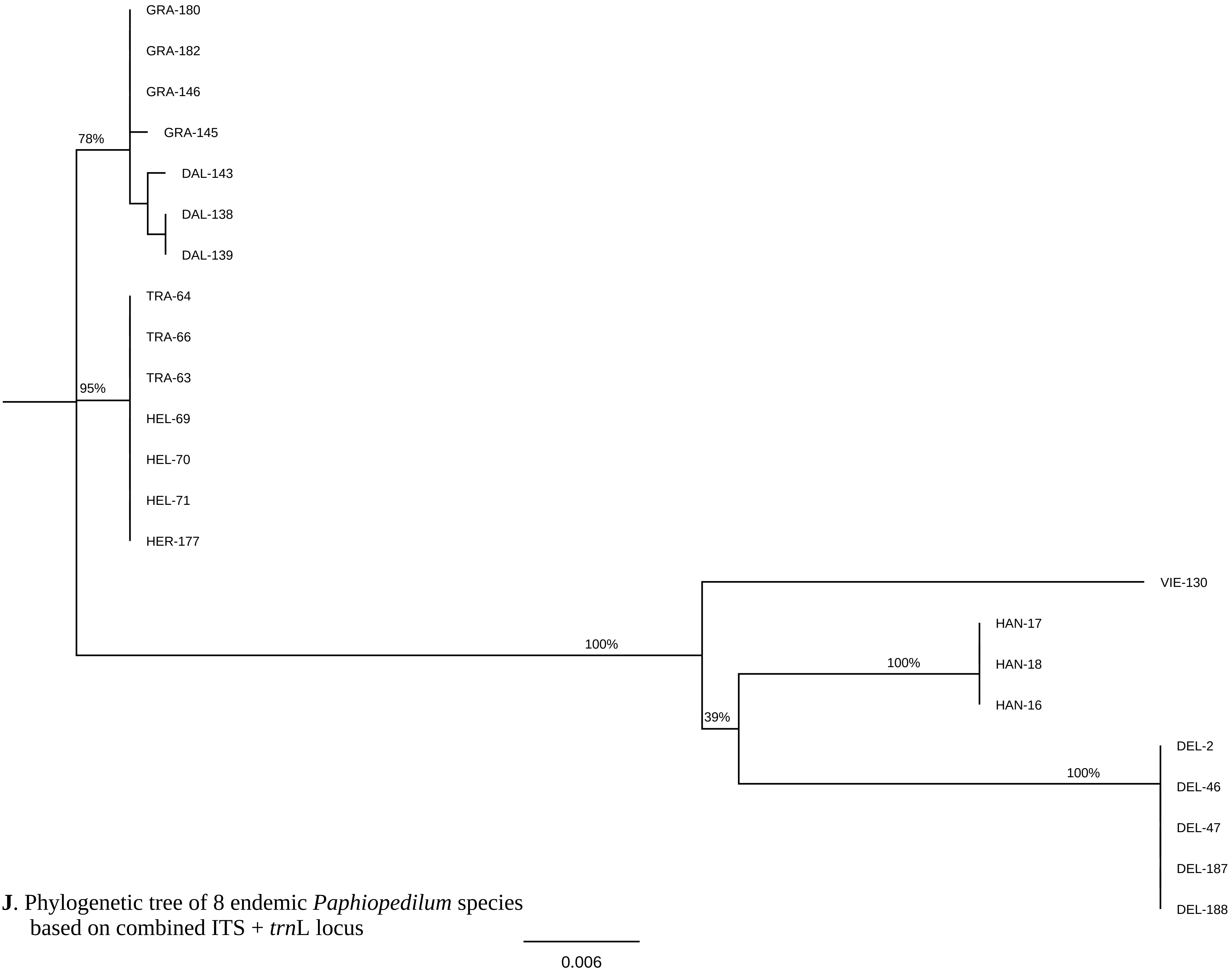

J. Phylogenetic tree of 8 endemic *Paphiopedilum* species based on combined ITS + *trn*L locus

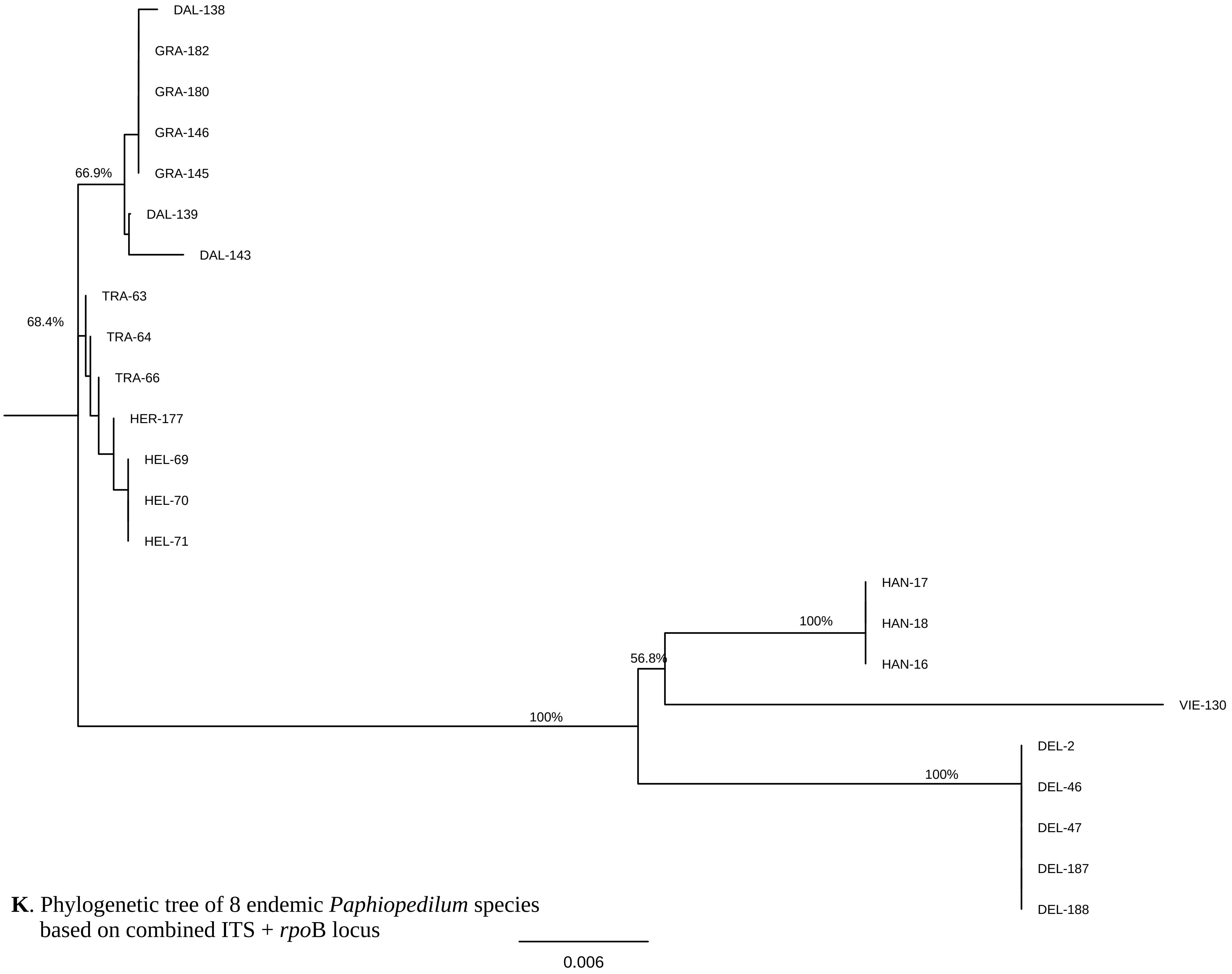

K. Phylogenetic tree of 8 endemic *Paphiopedilum* species based on combined ITS + *rpo*B locus

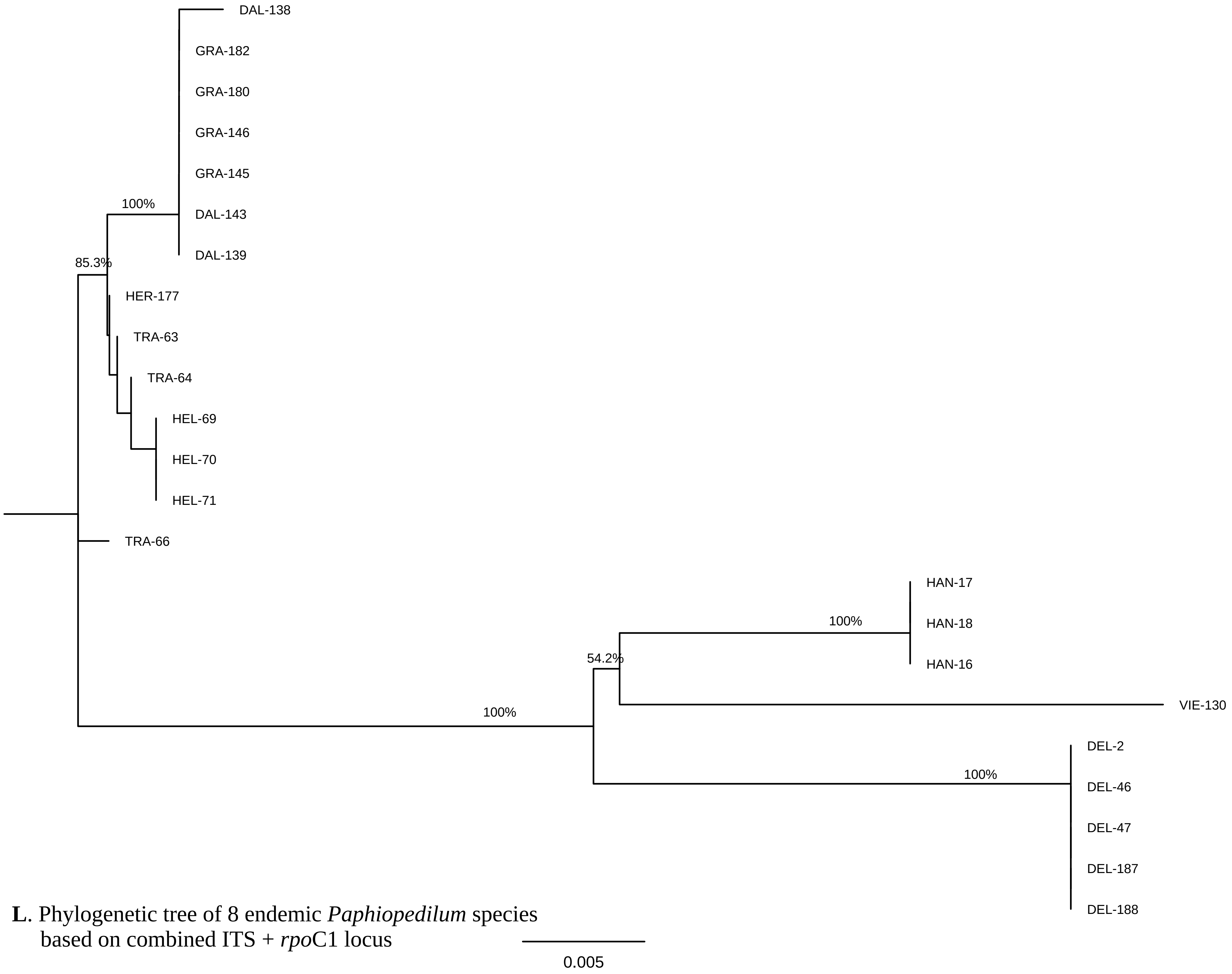

L. Phylogenetic tree of 8 endemic *Paphiopedilum* species based on combined ITS + *rpo*C1 locus
