# Supplementary material for: Genetic Diversity and Identification of Vietnamese Paphiopedilum Species Using DNA Sequences
Source: Biology (Basel). 2019 Dec 31;9(1):9. doi: 10.3390/biology9010009 (PMC7168009; doi:10.3390/biology9010009)
Supplement: Supplementary file 1 [file biology-09-00009-s001.zip › Supplementary Figure S2.pdf]

Figure S2. Estimates of evolutionary divergence between sequences of 8 endemic *Paphiopedilum* species based on 5 single sequences ITS, *mat*K, *trn*L, *rpo*B, *rpo*C1

ITS matrix		1	2	3	4	5	6	7	8	9	10	11	12	13	14	15	16	17	18	19	20	21	22	23
1	DEL-2
2	DEL-46	0.000
3	DEL-47	0.000	0.000
4	DEL-187	0.000	0.000	0.000
5	DEL-188	0.000	0.000	0.000	0.000
6	DAL-138	0.073	0.073	0.073	0.073	0.073
7	DAL-139	0.073	0.073	0.073	0.073	0.073	0.000
8	DAL-143	0.073	0.073	0.073	0.073	0.073	0.000	0.000
9	GRA-145	0.073	0.073	0.073	0.073	0.073	0.000	0.000	0.000
10	GRA-146	0.073	0.073	0.073	0.073	0.073	0.000	0.000	0.000	0.000
11	GRA-180	0.073	0.073	0.073	0.073	0.073	0.000	0.000	0.000	0.000	0.000
12	GRA-182	0.073	0.073	0.073	0.073	0.073	0.000	0.000	0.000	0.000	0.000	0.000
13	HAN-16	0.047	0.047	0.047	0.047	0.047	0.063	0.063	0.063	0.063	0.063	0.063	0.063
14	HAN-17	0.047	0.047	0.047	0.047	0.047	0.063	0.063	0.063	0.063	0.063	0.063	0.063	0.000
15	HAN-18	0.047	0.047	0.047	0.047	0.047	0.063	0.063	0.063	0.063	0.063	0.063	0.063	0.000	0.000
16	HEL-69	0.074	0.074	0.074	0.074	0.074	0.009	0.009	0.009	0.009	0.009	0.009	0.009	0.061	0.061	0.061
17	HEL-70	0.074	0.074	0.074	0.074	0.074	0.009	0.009	0.009	0.009	0.009	0.009	0.009	0.061	0.061	0.061	0.000
18	HEL-71	0.074	0.074	0.074	0.074	0.074	0.009	0.009	0.009	0.009	0.009	0.009	0.009	0.061	0.061	0.061	0.000	0.000
19	HER-177	0.069	0.069	0.069	0.069	0.069	0.005	0.005	0.005	0.005	0.005	0.005	0.005	0.056	0.056	0.056	0.000	0.000	0.000
20	TRA-63	0.069	0.069	0.069	0.069	0.069	0.005	0.005	0.005	0.005	0.005	0.005	0.005	0.056	0.056	0.056	0.000	0.000	0.000	0.000
21	TRA-64	0.069	0.069	0.069	0.069	0.069	0.005	0.005	0.005	0.005	0.005	0.005	0.005	0.056	0.056	0.056	0.000	0.000	0.000	0.000	0.000
22	TRA-66	0.069	0.069	0.069	0.069	0.069	0.005	0.005	0.005	0.005	0.005	0.005	0.005	0.056	0.056	0.056	0.000	0.000	0.000	0.000	0.000	0.000
23	VIE-130	0.072	0.072	0.072	0.072	0.072	0.084	0.084	0.084	0.084	0.084	0.084	0.084	0.054	0.054	0.054	0.084	0.084	0.084	0.077	0.077	0.077	0.077

**min**	**0.000**
**max**	**0.084**
**average**	**0.041**

matK matrix		1	2	3	4	5	6	7	8	9	10	11	12	13	14	15	16	17	18	19	20	21	22	23
1	DEL-2
2	DEL-46	0.000
3	DEL-47	0.000	0.000
4	DEL-187	0.000	0.000	0.000
5	DEL-188	0.000	0.000	0.000	0.000
6	DAL-138	0.021	0.021	0.021	0.021	0.021
7	DAL-139	0.021	0.021	0.021	0.021	0.021	0.000
8	DAL-143	0.023	0.023	0.023	0.023	0.023	0.002	0.002
9	GRA-145	0.019	0.019	0.019	0.019	0.019	0.005	0.005	0.007
10	GRA-146	0.019	0.019	0.019	0.019	0.019	0.005	0.005	0.007	0.000
11	GRA-180	0.019	0.019	0.019	0.019	0.019	0.005	0.005	0.007	0.000	0.000
12	GRA-182	0.019	0.019	0.019	0.019	0.019	0.005	0.005	0.007	0.000	0.000	0.000
13	HAN-16	0.012	0.012	0.012	0.012	0.012	0.030	0.030	0.031	0.028	0.028	0.028	0.028
14	HAN-17	0.012	0.012	0.012	0.012	0.012	0.030	0.030	0.031	0.028	0.028	0.028	0.028	0.000
15	HAN-18	0.012	0.012	0.012	0.012	0.012	0.030	0.030	0.031	0.028	0.028	0.028	0.028	0.000	0.000
16	HEL-69	0.020	0.020	0.020	0.020	0.020	0.006	0.006	0.008	0.001	0.001	0.001	0.001	0.029	0.029	0.029
17	HEL-70	0.020	0.020	0.020	0.020	0.020	0.006	0.006	0.008	0.001	0.001	0.001	0.001	0.029	0.029	0.029	0.000
18	HEL-71	0.020	0.020	0.020	0.020	0.020	0.006	0.006	0.008	0.001	0.001	0.001	0.001	0.029	0.029	0.029	0.000	0.000
19	HER-177	0.019	0.019	0.019	0.019	0.019	0.004	0.004	0.006	0.000	0.000	0.000	0.000	0.028	0.028	0.028	0.001	0.001	0.001
20	TRA-63	0.019	0.019	0.019	0.019	0.019	0.005	0.005	0.007	0.000	0.000	0.000	0.000	0.028	0.028	0.028	0.001	0.001	0.001	0.000
21	TRA-64	0.019	0.019	0.019	0.019	0.019	0.005	0.005	0.007	0.000	0.000	0.000	0.000	0.028	0.028	0.028	0.001	0.001	0.001	0.000	0.000
22	TRA-66	0.019	0.019	0.019	0.019	0.019	0.005	0.005	0.007	0.000	0.000	0.000	0.000	0.028	0.028	0.028	0.001	0.001	0.001	0.000	0.000	0.000
23	VIE-130	0.009	0.009	0.009	0.009	0.009	0.024	0.024	0.026	0.022	0.022	0.022	0.022	0.014	0.014	0.014	0.023	0.023	0.023	0.022	0.022	0.022	0.022

**min**	**0.000**
**max**	**0.031**
**average**	**0.013**

trnL matrix		1	2	3	4	5	6	7	8	9	10	11	12	13	14	15	16	17	18	19	20	21	22	23
1	DEL-2
2	DEL-46	0.000
3	DEL-47	0.000	0.000
4	DEL-187	0.000	0.000	0.000
5	DEL-188	0.000	0.000	0.000	0.000
6	DAL-138	0.029	0.029	0.029	0.029	0.029
7	DAL-139	0.029	0.029	0.029	0.029	0.029	0.000
8	DAL-143	0.029	0.029	0.029	0.029	0.029	0.005	0.005
9	GRA-145	0.032	0.032	0.032	0.032	0.032	0.007	0.007	0.007
10	GRA-146	0.029	0.029	0.029	0.029	0.029	0.005	0.005	0.005	0.002
11	GRA-180	0.029	0.029	0.029	0.029	0.029	0.005	0.005	0.005	0.002	0.000
12	GRA-182	0.029	0.029	0.029	0.029	0.029	0.005	0.005	0.005	0.002	0.000	0.000
13	HAN-16	0.017	0.017	0.017	0.017	0.017	0.029	0.029	0.029	0.032	0.029	0.029	0.029
14	HAN-17	0.017	0.017	0.017	0.017	0.017	0.029	0.029	0.029	0.032	0.029	0.029	0.029	0.000
15	HAN-18	0.017	0.017	0.017	0.017	0.017	0.029	0.029	0.029	0.032	0.029	0.029	0.029	0.000	0.000
16	HEL-69	0.029	0.029	0.029	0.029	0.029	0.005	0.005	0.005	0.002	0.000	0.000	0.000	0.029	0.029	0.029
17	HEL-70	0.029	0.029	0.029	0.029	0.029	0.005	0.005	0.005	0.002	0.000	0.000	0.000	0.029	0.029	0.029	0.000
18	HEL-71	0.029	0.029	0.029	0.029	0.029	0.005	0.005	0.005	0.002	0.000	0.000	0.000	0.029	0.029	0.029	0.000	0.000
19	HER-177	0.029	0.029	0.029	0.029	0.029	0.005	0.005	0.005	0.002	0.000	0.000	0.000	0.029	0.029	0.029	0.000	0.000	0.000
20	TRA-63	0.029	0.029	0.029	0.029	0.029	0.005	0.005	0.005	0.002	0.000	0.000	0.000	0.029	0.029	0.029	0.000	0.000	0.000	0.000
21	TRA-64	0.029	0.029	0.029	0.029	0.029	0.005	0.005	0.005	0.002	0.000	0.000	0.000	0.029	0.029	0.029	0.000	0.000	0.000	0.000	0.000
22	TRA-66	0.029	0.029	0.029	0.029	0.029	0.005	0.005	0.005	0.002	0.000	0.000	0.000	0.029	0.029	0.029	0.000	0.000	0.000	0.000	0.000	0.000
23	VIE-130	0.005	0.005	0.005	0.005	0.005	0.024	0.024	0.024	0.027	0.024	0.024	0.024	0.012	0.012	0.012	0.024	0.024	0.024	0.024	0.024	0.024	0.024

**min**	**0.000**
**max**	**0.032**
**average**	**0.016**

rpoB matrix		1	2	3	4	5	6	7	8	9	10	11	12	13	14	15	16	17	18	19	20	21	22	23
1	DEL-2
2	DEL-46	0.000
3	DEL-47	0.000	0.000
4	DEL-187	0.000	0.000	0.000
5	DEL-188	0.000	0.000	0.000	0.000
6	DAL-138	0.016	0.016	0.016	0.016	0.016
7	DAL-139	0.012	0.012	0.012	0.012	0.012	0.004
8	DAL-143	0.014	0.014	0.014	0.014	0.014	0.010	0.006
9	GRA-145	0.014	0.014	0.014	0.014	0.014	0.002	0.002	0.008
10	GRA-146	0.014	0.014	0.014	0.014	0.014	0.002	0.002	0.008	0.000
11	GRA-180	0.014	0.014	0.014	0.014	0.014	0.002	0.002	0.008	0.000	0.000
12	GRA-182	0.014	0.014	0.014	0.014	0.014	0.002	0.002	0.008	0.000	0.000	0.000
13	HAN-16	0.002	0.002	0.002	0.002	0.002	0.014	0.010	0.012	0.012	0.012	0.012	0.012
14	HAN-17	0.002	0.002	0.002	0.002	0.002	0.014	0.010	0.012	0.012	0.012	0.012	0.012	0.000
15	HAN-18	0.002	0.002	0.002	0.002	0.002	0.014	0.010	0.012	0.012	0.012	0.012	0.012	0.000	0.000
16	HEL-69	0.014	0.014	0.014	0.014	0.014	0.002	0.002	0.008	0.000	0.000	0.000	0.000	0.012	0.012	0.012
17	HEL-70	0.014	0.014	0.014	0.014	0.014	0.002	0.002	0.008	0.000	0.000	0.000	0.000	0.012	0.012	0.012	0.000
18	HEL-71	0.014	0.014	0.014	0.014	0.014	0.002	0.002	0.008	0.000	0.000	0.000	0.000	0.012	0.012	0.012	0.000	0.000
19	HER-177	0.016	0.016	0.016	0.016	0.016	0.003	0.003	0.011	0.000	0.000	0.000	0.000	0.013	0.013	0.013	0.000	0.000	0.000
20	TRA-63	0.014	0.014	0.014	0.014	0.014	0.002	0.002	0.008	0.000	0.000	0.000	0.000	0.012	0.012	0.012	0.000	0.000	0.000	0.000
21	TRA-64	0.014	0.014	0.014	0.014	0.014	0.002	0.002	0.008	0.000	0.000	0.000	0.000	0.012	0.012	0.012	0.000	0.000	0.000	0.000	0.000
22	TRA-66	0.014	0.014	0.014	0.014	0.014	0.002	0.002	0.008	0.000	0.000	0.000	0.000	0.012	0.012	0.012	0.000	0.000	0.000	0.000	0.000	0.000
23	VIE-130	0.006	0.006	0.006	0.006	0.006	0.019	0.015	0.017	0.017	0.017	0.017	0.017	0.004	0.004	0.004	0.017	0.017	0.017	0.020	0.017	0.017	0.017

**min**	**0.000**
**max**	**0.020**
**average**	**0.008**

rpoC1 matrix		1	2	3	4	5	6	7	8	9	10	11	12	13	14	15	16	17	18	19	20	21	22	23
1	DEL-2
2	DEL-46	0.000
3	DEL-47	0.000	0.000
4	DEL-187	0.000	0.000	0.000
5	DEL-188	0.000	0.000	0.000	0.000
6	DAL-138	0.011	0.011	0.011	0.011	0.011
7	DAL-139	0.007	0.007	0.007	0.007	0.007	0.004
8	DAL-143	0.007	0.007	0.007	0.007	0.007	0.004	0.000
9	GRA-145	0.007	0.007	0.007	0.007	0.007	0.004	0.000	0.000
10	GRA-146	0.007	0.007	0.007	0.007	0.007	0.004	0.000	0.000	0.000
11	GRA-180	0.007	0.007	0.007	0.007	0.007	0.004	0.000	0.000	0.000	0.000
12	GRA-182	0.007	0.007	0.007	0.007	0.007	0.004	0.000	0.000	0.000	0.000	0.000
13	HAN-16	0.009	0.009	0.009	0.009	0.009	0.011	0.007	0.007	0.007	0.007	0.007	0.007
14	HAN-17	0.009	0.009	0.009	0.009	0.009	0.011	0.007	0.007	0.007	0.007	0.007	0.007	0.000
15	HAN-18	0.009	0.009	0.009	0.009	0.009	0.011	0.007	0.007	0.007	0.007	0.007	0.007	0.000	0.000
16	HEL-69	0.007	0.007	0.007	0.007	0.007	0.004	0.000	0.000	0.000	0.000	0.000	0.000	0.007	0.007	0.007
17	HEL-70	0.007	0.007	0.007	0.007	0.007	0.004	0.000	0.000	0.000	0.000	0.000	0.000	0.007	0.007	0.007	0.000
18	HEL-71	0.007	0.007	0.007	0.007	0.007	0.004	0.000	0.000	0.000	0.000	0.000	0.000	0.007	0.007	0.007	0.000	0.000
19	HER-177	0.007	0.007	0.007	0.007	0.007	0.004	0.000	0.000	0.000	0.000	0.000	0.000	0.007	0.007	0.007	0.000	0.000	0.000
20	TRA-63	0.007	0.007	0.007	0.007	0.007	0.004	0.000	0.000	0.000	0.000	0.000	0.000	0.007	0.007	0.007	0.000	0.000	0.000	0.000
21	TRA-64	0.007	0.007	0.007	0.007	0.007	0.004	0.000	0.000	0.000	0.000	0.000	0.000	0.007	0.007	0.007	0.000	0.000	0.000	0.000	0.000
22	TRA-66	0.009	0.009	0.009	0.009	0.009	0.011	0.007	0.007	0.007	0.007	0.007	0.007	0.004	0.004	0.004	0.007	0.007	0.007	0.007	0.007	0.007
23	VIE-130	0.007	0.007	0.007	0.007	0.007	0.009	0.004	0.004	0.004	0.004	0.004	0.004	0.007	0.007	0.007	0.004	0.004	0.004	0.004	0.004	0.004	0.007

**min**	**0.000**
**max**	**0.011**
**average**	**0.005**

(Numbers of the first horizontal line were corresponding to numbers of the fisrt vertical colume that represent studied samples.

Numbers inside were estimated genetic distance between pairs of samples.

min: minimum genetic distance of matrix
max: maximum genetic distance of matrix
average: average genetic distance of matrix)
