# Supplementary material for: Genetic Diversity and Identification of Vietnamese Paphiopedilum Species Using DNA Sequences
Source: Biology (Basel). 2019 Dec 31;9(1):9. doi: 10.3390/biology9010009 (PMC7168009; doi:10.3390/biology9010009)
Supplement: Supplementary file 1 [file biology-09-00009-s001.zip › Supplementary Figure S4.pdf]

Figure S4. **Phylogenetic trees of 21 Vietnamese *Paphiopedilum* species based on *mat*K region using different methods.**

(A) Neighbor-joining method in MEGA
(B) Maximum Likelihood method in PAUP*
(C) Bayesian Inference method in MRBAYES
(D) Maximum Parsimony method in PAUP*
(E) Maximum Likelihoo method in MEGA
Color samples: identified species. Black samples: unidentified species)

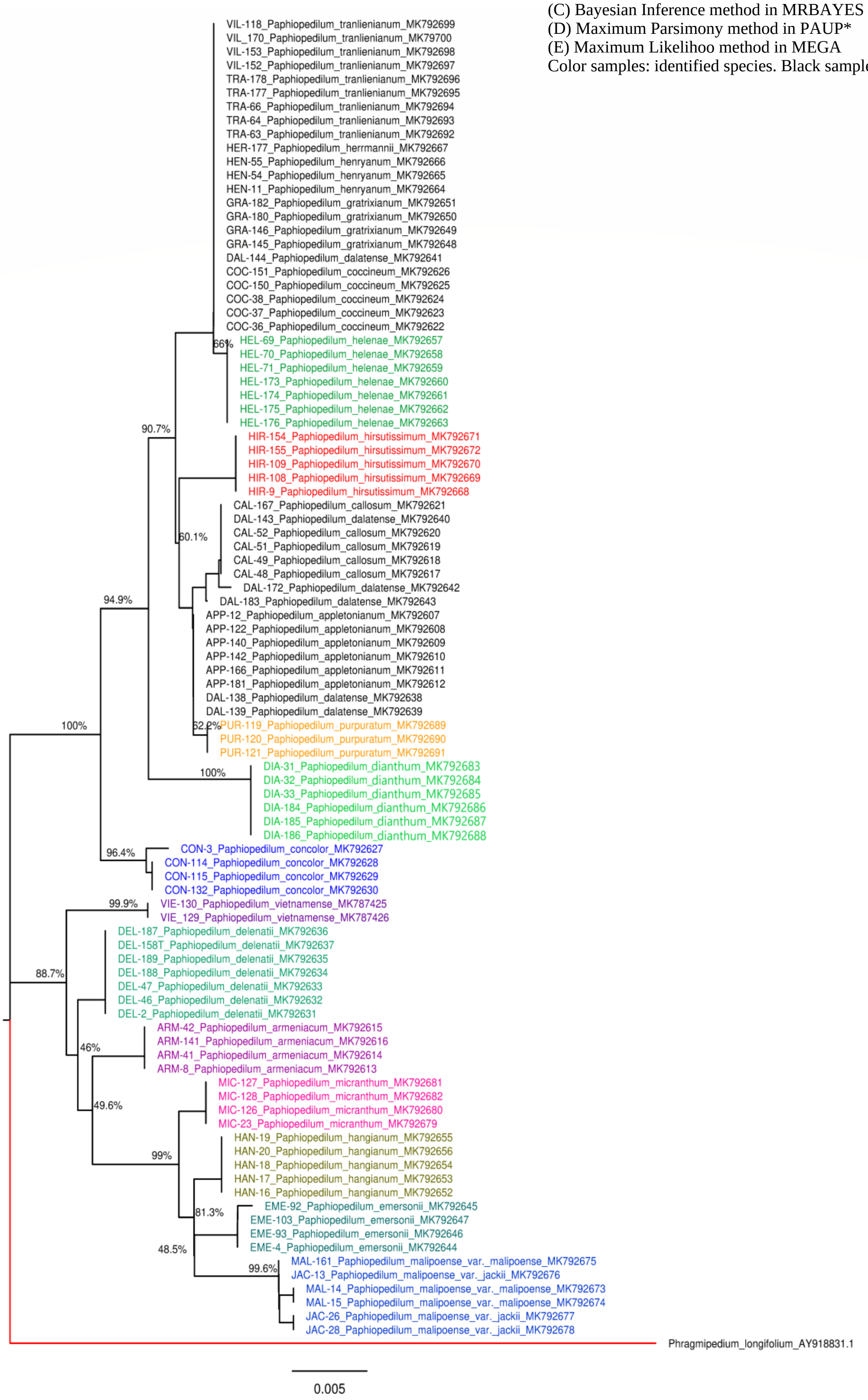

A. Neighbor-joining method in MEGA

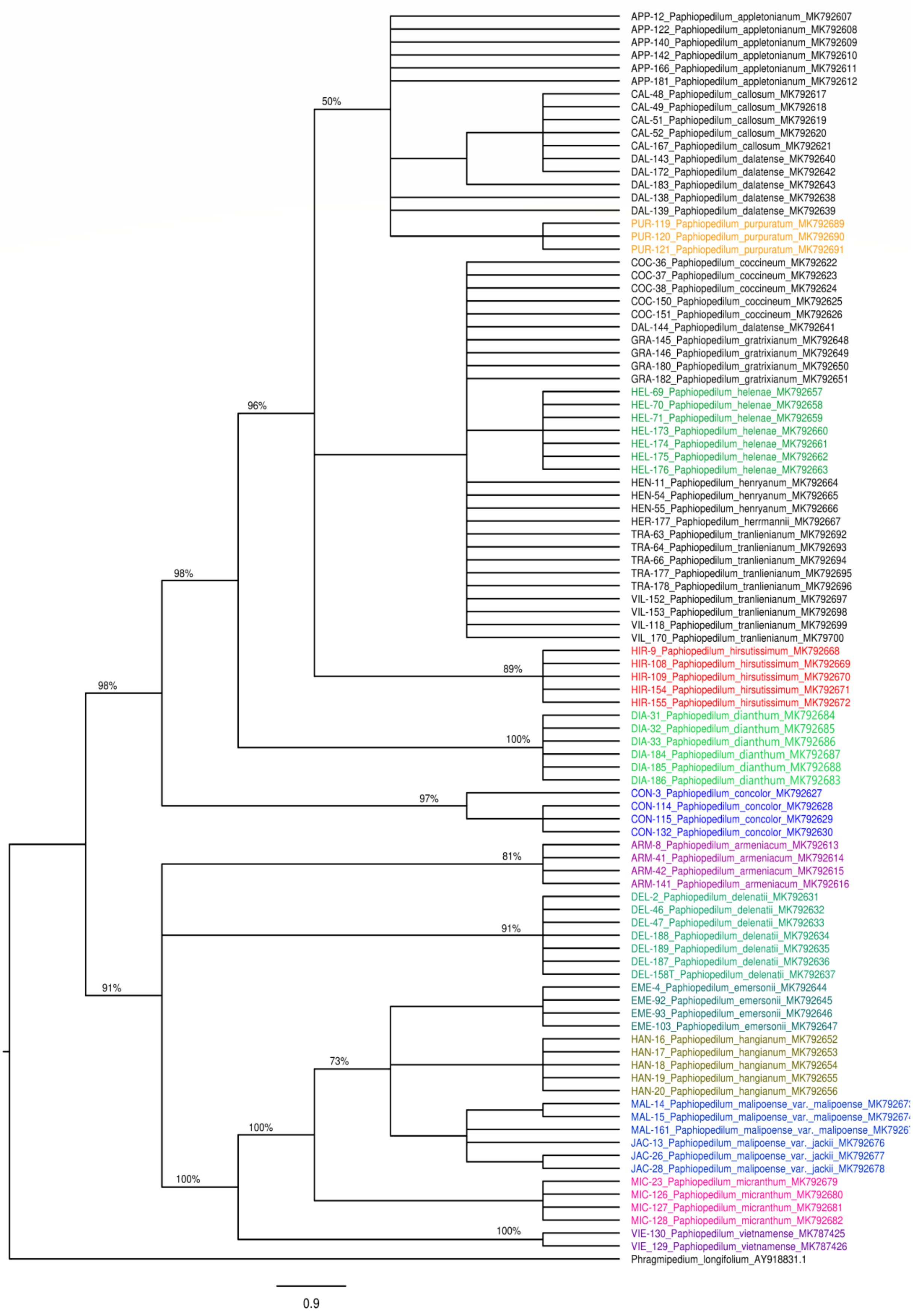

B. Maximum Likelihood method in PAUP*

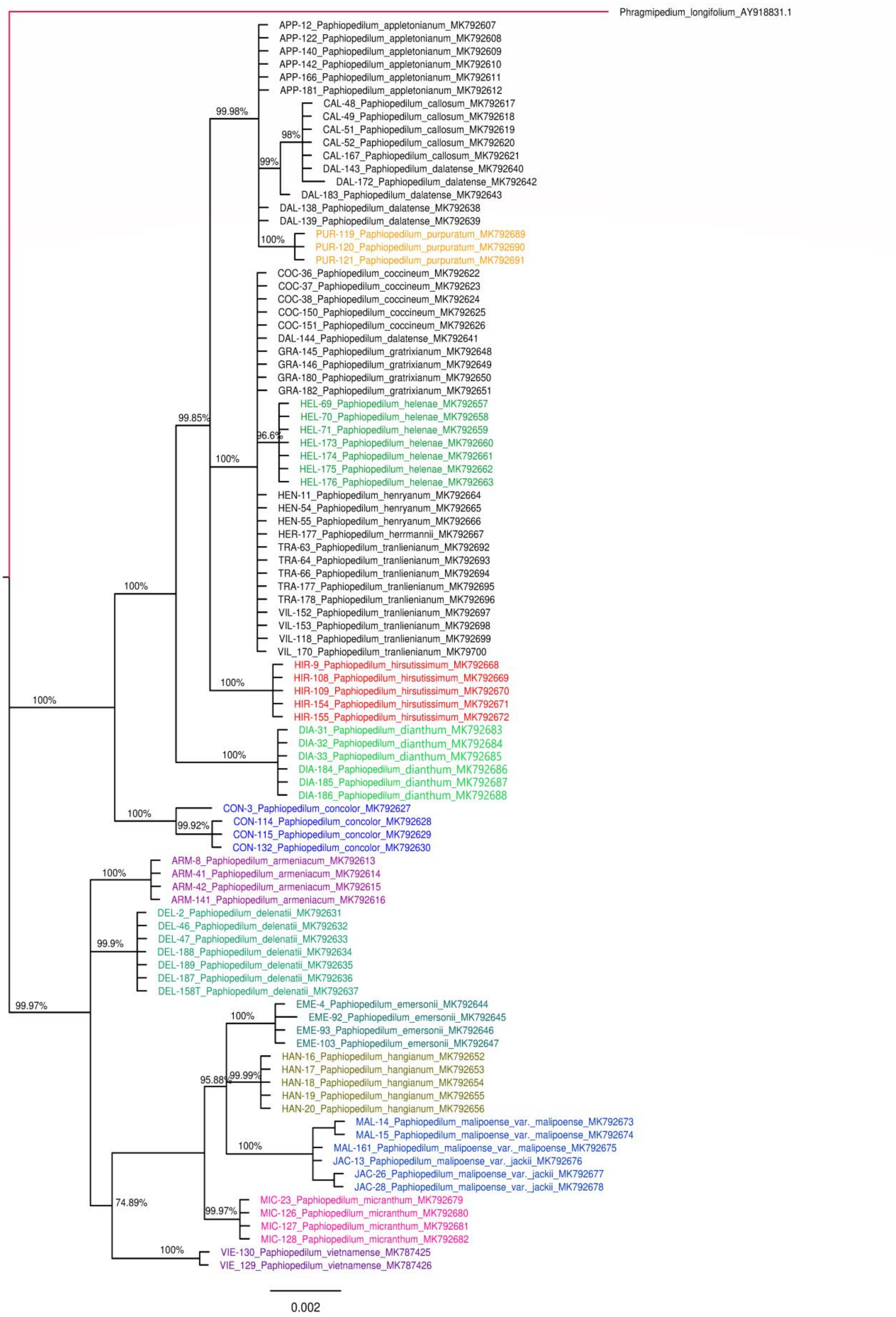

C. Bayesian Inference method in MRBAYES

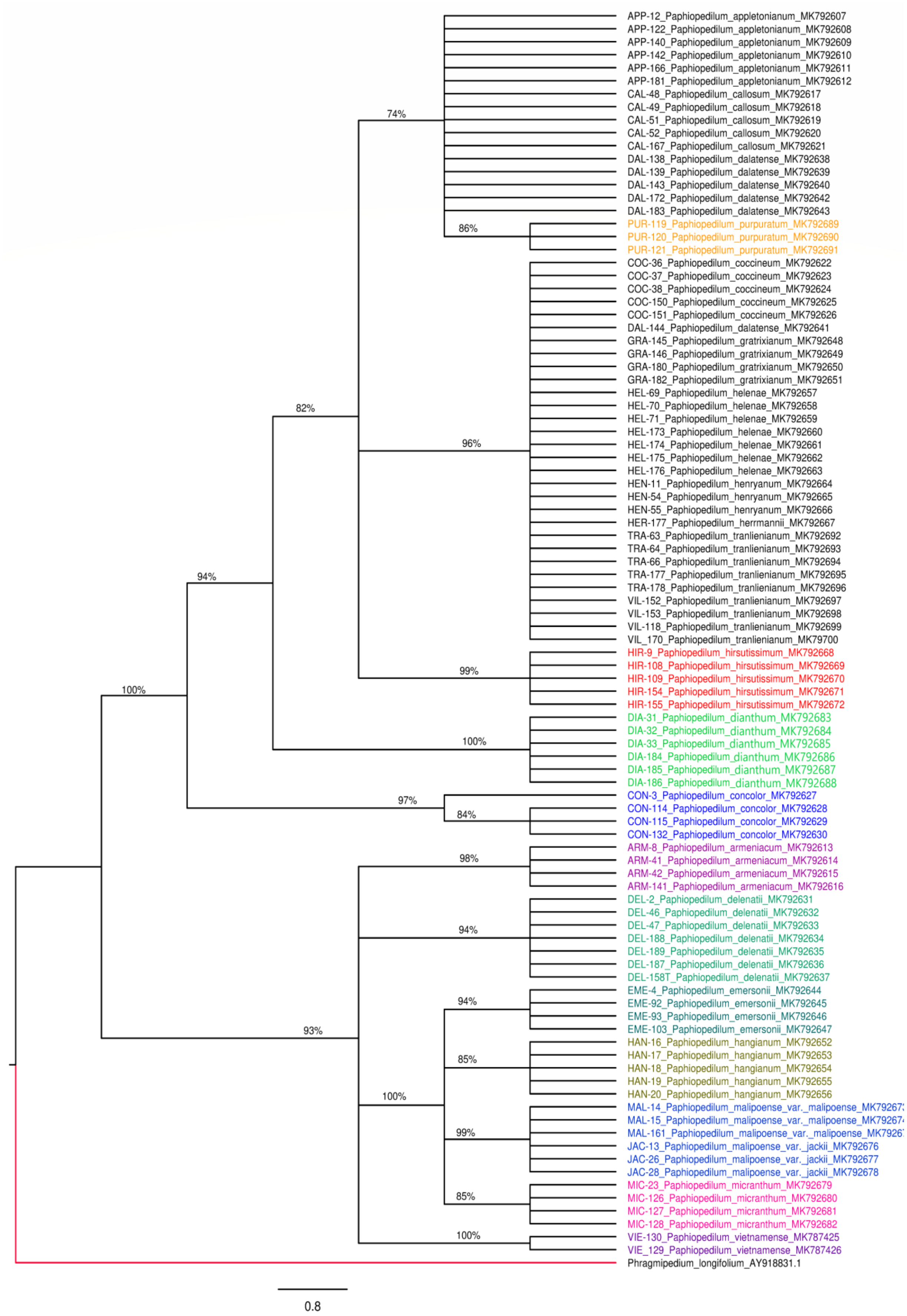

D. Maximum Parsimony method in PAUP*

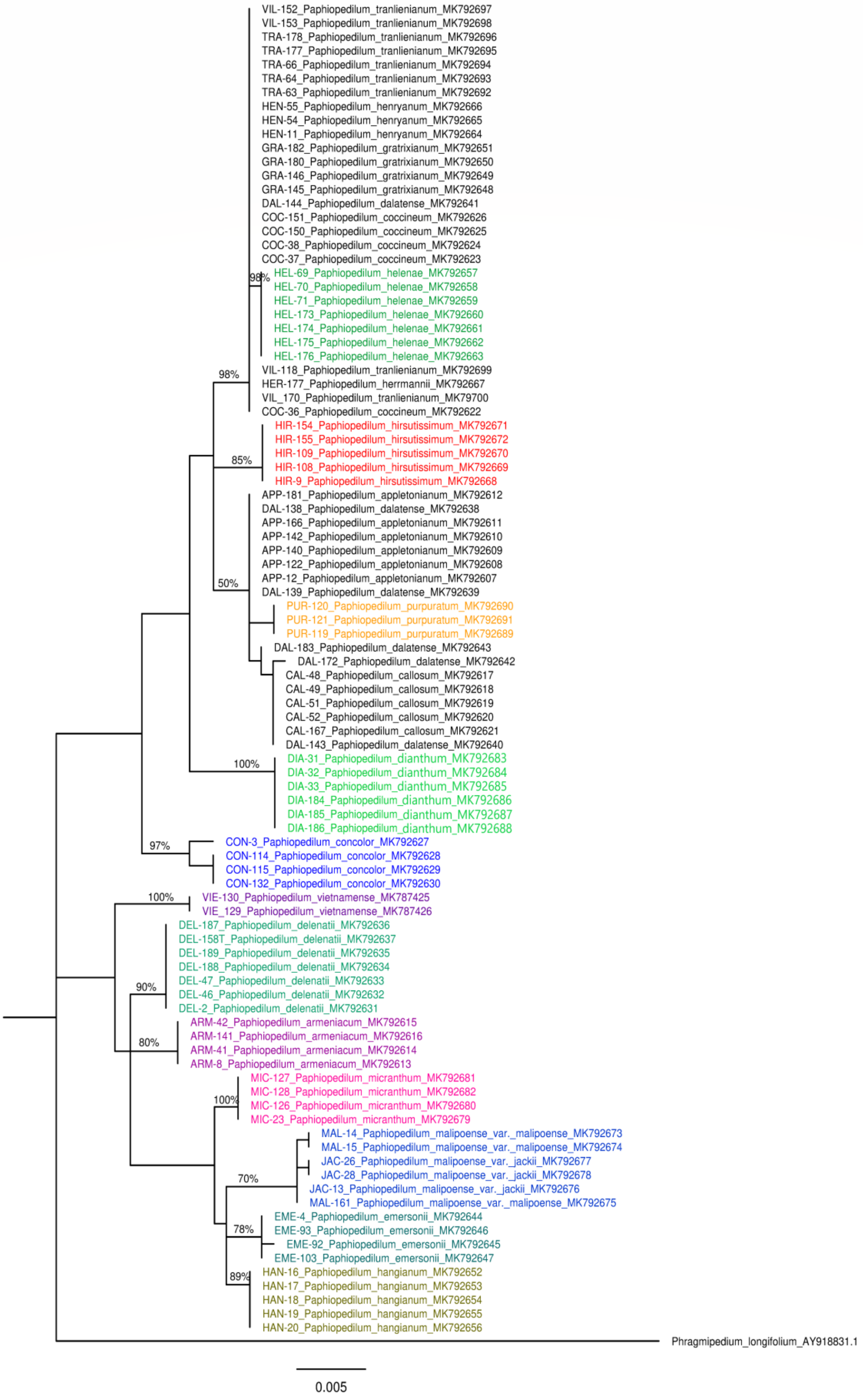

E. Maximum Likelihood method in MEGA
